# Using Likelihood-Free Inference to Compare Evolutionary Dynamics of the Protein Networks of H. pylori and P. falciparum

**DOI:** 10.1371/journal.pcbi.0030230

**Published:** 2007-11-30

**Authors:** Oliver Ratmann, Ole Jørgensen, Trevor Hinkley, Michael Stumpf, Sylvia Richardson, Carsten Wiuf

**Affiliations:** 1 Department of Public Health and Epidemiology, Imperial College London, London, United Kingdom; 2 Bioinformatics Research Center, University of Aarhus, Aarhus, Denmark; 3 Institut für Integrative Biologie, ETH Zürich, Zürich, Switzerland; 4 Theoretical Genomics Group, Centre for Bioinformatics, Division of Molecular Biosciences, Imperial College London, London, United Kingdom; 5 Institute of Mathematical Sciences, Imperial College London, London, United Kingdom; 6 Centre for Biostatistics, Imperial College London, London, United Kingdom; 7 Molecular Diagnostic Laboratory, Aarhus University Hospital, Aarhus, Denmark; ETH Zürich, Switzerland

## Abstract

Gene duplication with subsequent interaction divergence is one of the primary driving forces in the evolution of genetic systems. Yet little is known about the precise mechanisms and the role of duplication divergence in the evolution of protein networks from the prokaryote and eukaryote domains. We developed a novel, model-based approach for Bayesian inference on biological network data that centres on approximate Bayesian computation, or likelihood-free inference. Instead of computing the intractable likelihood of the protein network topology, our method summarizes key features of the network and, based on these, uses a MCMC algorithm to approximate the posterior distribution of the model parameters. This allowed us to reliably fit a flexible mixture model that captures hallmarks of evolution by gene duplication and subfunctionalization to protein interaction network data of Helicobacter pylori and Plasmodium falciparum. The 80% credible intervals for the duplication–divergence component are [0.64, 0.98] for H. pylori and [0.87, 0.99] for P. falciparum. The remaining parameter estimates are not inconsistent with sequence data. An extensive sensitivity analysis showed that incompleteness of PIN data does not largely affect the analysis of models of protein network evolution, and that the degree sequence alone barely captures the evolutionary footprints of protein networks relative to other statistics. Our likelihood-free inference approach enables a fully Bayesian analysis of a complex and highly stochastic system that is otherwise intractable at present. Modelling the evolutionary history of PIN data, it transpires that only the simultaneous analysis of several global aspects of protein networks enables credible and consistent inference to be made from available datasets. Our results indicate that gene duplication has played a larger part in the network evolution of the eukaryote than in the prokaryote, and suggests that single gene duplications with immediate divergence alone may explain more than 60% of biological network data in both domains.

## Author Summary

The importance of gene duplication to biological evolution has been recognized since the 1930s. For more than a decade, substantial evidence has been collected from genomic sequence data in order to elucidate the importance and the mechanisms of gene duplication; however, most biological characteristics arise from complex interactions between the cell's numerous constituents. Recently, preliminary descriptions of the protein interaction networks have become available for species of different domains. Adapting novel techniques in stochastic simulation, the authors demonstrate that evolutionary inferences can be drawn from large-scale, incomplete network data by fitting a stochastic model of network growth that captures hallmarks of evolution by duplication and divergence. They have also analyzed the effect of summarizing protein networks in different ways, and show that a reliable and consistent analysis requires many aspects of network data to be considered jointly; in contrast to what is commonly done in practice. Their results indicate that duplication and divergence has played a larger role in the network evolution of the eukaryote P. falciparum than in the prokaryote H. pylori, and emphasize at least for the eukaryote the potential importance of subfunctionalization in network evolution.

## Introduction

Genomic sequence data provides substantial evidence for the abundance of duplicated genes in all organisms surveyed: at least 40% of genes in two prokaryotes [1,2] and 15%–90% of genes in eukaryotes [3–5] appear to be products of gene duplication. This suggests that gene duplication is a key mechanistic driving force behind the evolution of complex organisms [6]. In particular, the fact that the number of interactions shared by paralogous proteins decreases with sequence similarity in Saccharomyces cerevisiae [7,8] indicates that gene duplication might shape the topology of protein networks.

In theory, the evolutionary fate of gene duplicates can differ: (D1) one copy may become silenced (nonfunctionalization); (D2) both copies are very similar in sequence, and one is functionally redundant to the other [9]; (D3) both copies are mutationally compromised, and one or more subfunctions of the single progenitor are altered (subfunctionalization); or (D4) one copy may acquire a novel function preserved by natural selection, while the other copy retains the original function (neofunctionalization). The strength of (D3) is that it does not rely on the sparse occurrence of beneficial mutations, but on more frequently occurring loss-of-function mutations in regulatory regions [10,11]. Alternatively, based mostly on the assumption that the number of protein pairs that may acquire a novel function is large, several studies [7,12,13] promoted the relative importance of (D4), as well as the formation or degeneration of functional links between proteins in general (link turnover).

The structure of protein interaction networks (PINs) derives from multiple stochastic processes over evolutionary time scales, and a number of mechanisms have been proposed to capture aspects of network growth [12,14–16]. These models correspond to our limited understanding of network evolution, and there is no consensus as to which mechanisms are required to produce “realistic” models for biological PINs [17]. What is required is to be able to fit to biological network data a model (or mixture of models) of growing networks that reproduce more accurately the properties of real biological networks than simple preferential attachment [18] or duplication models [16]. For duplication–attachment models of network growth, Wiuf et al. [19] developed a full likelihood approach; this class of models, however, does not adequately explain the structure of most biological network data.

The analysis of PINs is notoriously difficult because measurements of PINs are subject to considerable levels of noise [20,21], and in their present guise, offer only an incomplete description of the true interaction network [22]. Interaction datasets are also highly averaged, not only over technical aspects such as the experimental protocol, but also over the precise cellular conditions under which interactions take place, interaction strength, and individual variation.

In this work, we develop an approximate, likelihood-free Monte-Carlo inference technique based on approximate Bayesian computation (ABC) [23–26] for inference on biological protein network data. Previously, an approximate composite likelihood approach has been proposed, using only the degree sequence to test whether or not simple scale-free models offer an adequate description of PIN data [27]. Owing to the complexity of PINs, we take multiple features of the data into account, which characterize PINs more fully. Our likelihood-free approach allows us to reliably compare more complex models of network evolution in order to study the relative importance of aspects of gene duplication and subsequent interaction divergence in prokaryotic and eukaryotic network evolution. Within the limits of the model and the available data, we find evidence for different dynamics in PIN evolution between the prokaryotic and eukaryotic domains as represented by H. pylori and P. falciparum, respectively.

The degree sequence [18], as well as the frequency profile of motif counts [28] are widely used to analyze protein network data. Our analysis shows that the degree sequence barely captures evolutionary footprints of PINs relative to other statistics. It also suggests that motif counts are extremely variable over the modelled evolutionary history of PINs, and thus inference based on these alone is fragile. Only the simultaneous analysis of many global aspects of PIN data rendered our evolutionary study credible and consistent.

## Results/Discussion

### Modelling the Evolution of Protein Networks

To study the relative importance of aspects of duplication divergence in network evolution between different domains, we simulated the evolutionary history of PINs with a mixture of duplication divergence with parent–child attachment (DDa) and preferential attachment (PA); see Box 1. At each step, the network either grows according to DDa with probability 1 − α or PA with probability α. More precisely, let *G_t_* be a network with *t* nodes (proteins), *v* a new node, *u* a randomly chosen parent node in *G_t_*, *δ*_Div_ the divergence probability, *δ*_Att_ the parent–child attachment probability, and let *θ* = (*δ*_Div_, *δ*_Att,_ *α*). Then the probability of *G_t_*_+1_ conditional on *G_t_* and *u* is

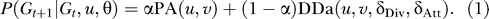

**Box 1. Glossary of Randomly Growing Graphs**

PINs can be described as graphs (Figure 7), which contain a set of **nodes** representing proteins with observed interactions, and **edges** representing observed interactions between proteins. Here, we focus on **undirected, unweighted, binary interactions** representing physical or indirect interaction under possibly different experimental conditions. **Randomly growing graphs** model the long-term, undirected evolution of protein networks; here, we consider two simple, **local growth mechanisms** that add a single node to the network at a time.

**Duplication Divergence with parent–child attachment** (DDa) [14,58] features a node duplication step followed immediately by an interaction divergence step. At each step (Figure 7), a parent node (orange) is randomly chosen, and its edges are duplicated. Each of the parental or duplicated edges (purple) is then lost (i.e., diverges) with probability *δ*_Div_; but for each parental edge, either the parental or the duplicated one must be retained after divergence. An interaction of the parent node with its child (blue, as indicated by the blue arrow) is given probability *δ*_Att_. DDa also generates nodes with no interactions; we impose that DDa does not generate nodes with no interactions.

**Preferential Attachment** (PA). A new node (purple) is added to the network, and attached to an existing node (orange) with probability proportional to the node degree (number of black edges per node).

The final steps in the graphs display possible realizations of the DDa and PA mechanisms, respectively.

The terms PA(*u*, *v*) and DDa(*u*, *v*, *δ*_Div,_ *δ*_Att_) correspond to the probabilities of moving to the new configuration under PA and DDa, respectively. They are explained in Box 1 and defined fully in Protocol S1. By repeated application of the mechanism in Equation 1, we grew PINs to the approximate number of open reading frames in the respective genomes (H. pylori: 1,500, and P. falciparum: 5,300).

We chose this mixture evolution model for a number of reasons. DDa agrees with aspects of genome evolution by gene duplication [29]. Several studies [7,8,10,30] found a rapid divergence of the interaction profiles of duplicate genes, indicating that duplication and subsequent divergence might be adequately modelled in a single step. Importantly, DDa may relate to subfunctionalization [31]: as a rule, at least one edge disappears, and the duplicates share the pleiotropy of the parent node [10,32]. Also, the model does not disagree with purifying selection that maintains the ancestral function at both duplicates [9,33,34], because, occasionally, all ancestral edges are retained.

The second component of the mixture model, first introduced in [18], is a generic local growth mechanism based on PA that may explain some characteristics of networks, in particular the approximate power-law decay of the node degrees. In the present context, it captures effects of network growth which are not specifically related to (D1–D3). Such effects are likely present in network evolution; Middendorf et al. [35] showed that PINs simulated by DDa alone may underrepresent tree-like subgraphs, whereas these are more accurately generated by PA. Also, horizontal gene transfer is a major force in prokaryote evolution. It is plausible to model such transfer with an attachment process, although no particular model has been proposed in the literature.

Overall, in the mixture model (Equation 1), network evolution proceeds by repeated node addition. Apart from rate homogeneity over all proteins, there are thus no further assumptions on the evolutionary clock of our model; a property that is particularly desirable because evolutionary events such as duplication or interaction divergence are generally unavailable or difficult to estimate reliably. Since link turnover is suspected to operate on a different time scale than duplication divergence, extending the model (Equation 1) with preferential link rewiring [12] would imply further assumptions on the evolutionary clock; potentially, phylogenetic data could help to fit such birth and death models of network evolution.

The evolution parameters are abstract quantities that subsume a number of complex biological processes [36]. The parameter *δ*_Div_ may, for example, relate to mutations and insertions or deletions on the sequence level, but also to novel posttranslational modifications or translocations into a different cellular compartment of one interaction partner. Notably, *δ*_Div_ is associated with immediate divergence and thus differs from divergence probabilities obtained from sequence data, since the latter are usually inferred over a time interval [37]. The parameter *δ*_Att_ represents the probability of link formation between duplicates. In this study, the mixture parameter α is of particular interest; we ask whether and to what extent, despite high incompleteness, the PIN topology of representatives from the prokaryotic and eukaryotic domains contain evolutionary footprints that may be related to a model that captures hallmarks of network evolution by (D1–D3).

### Modelling PIN Datasets

To account for incomplete data, random subnetworks of order *N* are chosen from the simulated networks that are grown to approximately the number of open reading frames in the respective genomes. Here, *N* is the number of proteins with observed interactions in the two datasets (H. pylori: 675 and P. falciparum: 1,271). The PIN datasets generated by Equation 1 and subsequent subsampling are dominated by stochastic effects (Figure S1); nevertheless, different parameters leave distinguishable imprints on simulated PINs (Figure S2).

### Likelihood-Free Inference of Protein Networks

The Bayesian paradigm is a powerful probabilistic framework for making inference on complex stochastic systems and allows all sources of uncertainty to be accounted for [38]. We applied this paradigm to estimate the posterior density *p(θ*| D℘) of *θ*, given a real PIN dataset D℘. Bayes' Theorem relates *p*(*θ*| D℘) to the likelihood *p*( D℘|*θ*) and the prior of *θ*, *p*(*θ*), via

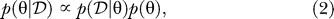
where ∝ denotes “proportional to.” In the absence of substantial prior information on *θ*, we use a uniform prior. The increased flexibility of Equation 1 comes at a computational cost and prohibits likelihood calculations that have been formalized by Wiuf et al. [19] for only very simple evolution models.

ABC confers computational tractability by circumventing the problem of evaluating the likelihood directly [23–26] and relies instead on the simulation of networks and the computation of network summaries. All ABC algorithms have in common to approximate first the data D℘ by a set of summaries S℘_ D℘_, for example ND and DIA (see Box 2 for a glossary of summary statistics and their abbreviations in the text) in the case of protein networks, and then proceed through several steps to sample parameter values from an approximate posterior density; see Materials and Methods for details. One approach is to sample from the prior density (noninformative in our case) and accept the proposed value, given that certain criteria are fulfilled. However, as suggested by Figure S2, only a small range of parameter values generate data with summaries close to S℘_ D℘_. Consequently, we anticipate that generating candidate parameters from the prior will be highly inefficient.

**Box 2. Glossary of Graph Summaries**

**CC** Average Cluster Coefficient, mean probability that two neighbours of a node are themselves neighbours.

**Degree** The number of edges associated with a node.

**DIA** Diameter, the longest minimum path among pairs of nodes in a connected component of the network.

**Distance** The distance between nodes *i* and *j* is the minimum number of edges that have to be visited to reach *j* from *i*.

**FRAG** Fragmentation, the percentage of nodes not in the largest connected component.

**ND** Node Degree Distribution or Degree Sequence, *p*(*nd* = *k*), the percentage of nodes with degree *k* in a network.

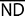
 Average Node Degree, the mean degree of a network.

**Order** The number of nodes in a network.

**PL** Average Path Length, the average distance of all node pairs in a connected component in the network.

**Size (R)** The number of edges in a network.

**TRIA** Number of Triangles, the number of 3-cycles in the network.

**WR** Within-Reach Distribution, *p*(*wr* ≤ *k*), the mean probability of how many nodes are reached from one node within distance *k* in the network.

Likelihood-free inference (LFI) within Markov Chain Monte Carlo (MCMC) [25] improves efficiency of standard ABC by exploiting knowledge of the current parameter value to make an educated guess on the next one. The details of algorithm ABC-MCMC are outlined in Material and Methods. It is guaranteed to eventually generate a series of correlated samples from

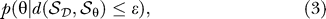
where *ɛ* is the tolerance according to the distance function *d*, and S℘*_θ_* is the set of summary statistics calculated on simulated data with parameter *θ*. If *ɛ* is large, then Equation 3 will roughly equal the prior. On the other hand, if *ɛ* is very small, then the estimator (Equation 3) is too variable. In the latter case, MCMC may become inefficient or even fail [25,26]. If *ɛ* is small and the set of summaries captures all aspects of the protein network sufficiently well, then

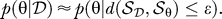



In order to achieve an approximation of the posterior for inference on protein networks, we modified ABC-MCMC to our algorithm LFI; see also Materials and Methods.

#### Averaging over an ensemble of PIN data during burn-in.

LFI compares summaries of the observed dataset with mean summaries S℘ of an ensemble of simulated PINs at each iteration of the algorithm during the burn-in phase, i.e., the first 800 iterations in this study. Since mean summaries over larger ensembles have reduced variance (Figure S3), LFI initially accepts parameters that are disproportionally close to the posterior mode. Consequently, algorithm LFI enables to burn in rapidly, enhancing computational efficiency. Previous studies [39,40] used averaging in similar numerical methods to efficiently approximate the maximum of the likelihood. We found that averaging summary statistics over 50 generated PINs is sufficient to burn in rapidly.

#### Tempering of *ɛ* and Σ.

LFI within MCMC is often prone to get stuck or to sit in the tails of the distribution [26]; essentially because the likelihood ratio within MCMC is coarsely approximated by either zero or one. To avoid the chain getting stuck, we adopted a tempering scheme [41] on the threshold *ɛ*. That is, during the burn-in phase, acceptance of parameters is controlled by a decreasing sequence of thresholds until a minimal, preset value *ɛ*_min_ is reached. To avoid the chain sitting in tails, it is in our case sufficient to temper the proposal variance Σ. See Materials and Methods for further details.

#### Summarizing aspects of protein networks.

**Figure 1:**
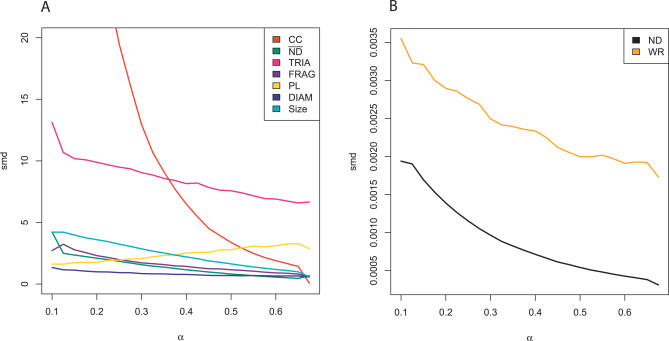
Choosing Appropriate Summaries with a Characterization of Genuine Change The standardized mean gradient smd is plotted as a function of *α.* Fifty networks corresponding to H. pylori (grown to 1,500 nodes and subsampled to 675) were generated as described in the text with *θ* ∈ [0.1, 0.7] × [0, 0.5] × [0.1, 0.6] in steps of 0.025; all mean summaries were computed for each *θ*. The marginal smd(α) is plotted for (A) summary statistics and (B) summary distributions. Together with cv in Figure S4, smd characterizes the sensitivity and variability of single summary statistics on simulated data. All summaries except ND have smd not close to zero, whereas TRIA, FRAG, and CC are extremely variable. Results for the other two parameters are very similar (unpublished data). The range of CC was truncated for display purposes.

Choosing appropriate summary statistics is central to any method approximating the true likelihood. This choice is governed by the principle that useful summaries should be sensitive to genuine changes in real PINs. Briefly, we characterized genuine changes by comparing the standardized mean derivative (smd) of a summary smd(*θ*), and the variation cv(*θ*) of the summary for varying values of *θ*. As further described in Materials and Methods, both smd(*θ*) and cv(*θ*) are scaled and use the same distance measure across summaries, so that our analysis allows us to compare summaries one by one. Except for ND, smd was not close to 0, and highest for CC and TRIA. Figure 1 illustrates this for the mixture parameter *α*. CC and TRIA, as well as FRAG, had the greatest variability, whereas ND showed almost no random fluctuations (see Figure S4). Taken together, this indicates that relative to other statistics, motif counts are extremely variable for fixed *θ*, and that ND has very limited power to detect genuine changes in *θ*. We derived a novel distributional statistic, the within-reach distribution (WR), that is more sensitive to changes in *θ* than ND; see Materials and Methods. Indeed, WR conveyed twice as much information as ND for the mixture parameter *α*; see Figure 1B.

#### Stringent distance function.

**Figure 2:**
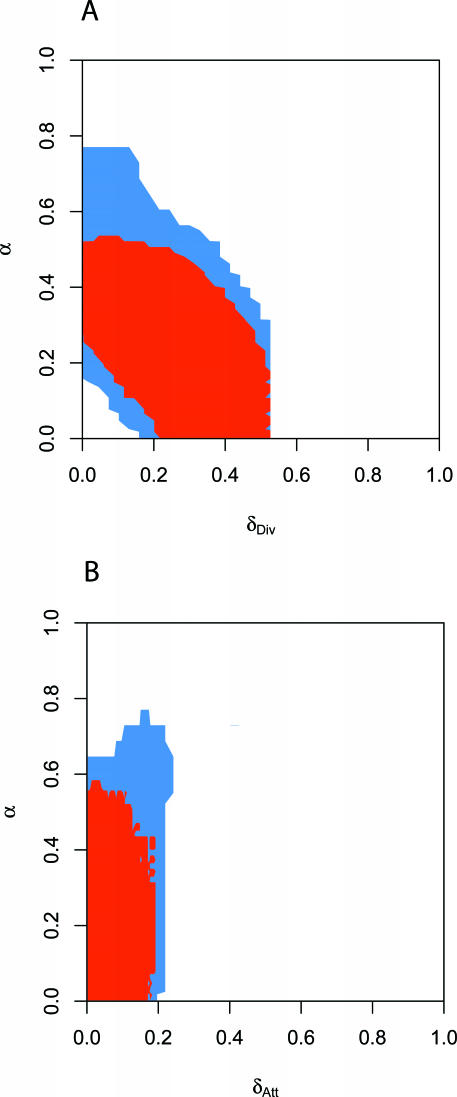
Comparing Distance Functions on a Set of Summaries. To compare different distance functions on sets of summaries, we analyzed the two-dimensional posterior support of *θ* for the H. pylori PIN dataset. (A) *α* versus *δ*_Div_ and (B) *α* versus *δ*_Att_. Using LFI with the set of summaries WR + DIA + CC + 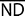 + FRAG, we recorded after burn-in the accepted parameters when each mean summary differed from the observed summary within the respective thresholds *ɛ_k_*,_min_ (*d*_∩_, red), and when the sum of these differences did not exceed the sum Σ*_k_ɛ_k_*,_min_ of these thresholds (*d*_Σ_, blue). In both cases, we used an average of shifted histograms to estimate the two-dimensional posterior support. When using *d*_∩_, the posterior support was more restricted, prompting us to use *d*_∩_ in LFI.

LFI is sensitive to the particular type of distance function *d* on a set of summaries. Often, a linear combination of standardized summaries is used [24]; instead, as detailed in Materials and Methods, we require that each summary over simulated PIN data is sufficiently close to the respective observed summary. Figure 2 shows that posterior support obtained by a simple linear combination differed not only in scale, but also in shape from the one obtained by our more stringent approach *d*_∩_.

In summary, for inference on protein networks our results suggest that

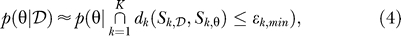
where each *S_k_* denotes the *k*th summary in S℘ and *S*_*k*,*θ*_ has non-zero smd(θ) and moderate cv(*θ*) over the range of *θ*, and all S*_k,θ_* are averaged during burn-in.

### The Role of Aspects of Duplication and Divergence in the Network Evolution of H. pylori and P. falciparum

Our evolutionary analysis of real PIN datasets centres on a comparison of two representatives from the prokaryotic and eukaryotic domain. We obtained descriptions of the PINs of H. pylori and P. falciparum from the Database of Interacting Proteins (http://dip.doe-mbi.ucla.edu). We first investigated LFI with different sets of summaries on simulated data as outlined in Protocol S1; based on the test results, we selected the set of summaries WR + DIA + CC + 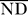 + FRAG for LFI.

**Figure 3:**
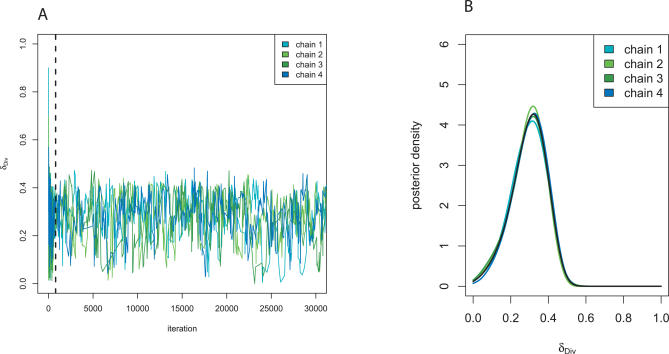
LFI on the H. pylori PIN For the H. pylori PIN dataset, four MCMC chains were run for 75,000 iterations according to LFI based on the summaries WR + DIA + CC + 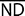 + FRAG. (A) The four chains for the parameter *δ*_Div_ ∈ [0,1] over the first 30,000 iterations. During burn-in, the chains moved quickly from overdispersed starting values and converged toward the same narrow support. Before iteration 800 (vertical line), *ɛ* was cooled to the minimal temperature; thereafter, accepted parameters were recorded, representing samples from the approximate posterior (Equation 4). (B) Accepted parameters after convergence were pooled over the four chains and used to estimate the posterior density. For *δ*_Div_, the marginal posterior is displayed (black line); in addition, posteriors were calculated for each chain and are overlaid, showing that the four sets of posterior samples overlapped well.

We successfully applied LFI on the H. pylori PIN. Figure 3 presents the MCMC chains for the divergence parameter *δ*_Div_ ∈ [0,1], and the estimated posterior *p(δ*_Div_| D℘). Similar good convergence was obtained for the attachment probability *δ*_Att_ and the mixture parameter *α*, and the 80% credible intervals (i.e., the inner range of values of a random variable that attains 80% posterior mass) are presented in Table 1. Technically important, the Markov chain resulting from algorithm LFI did not get stuck and did not sit in the tails for relatively small threshold values *ɛ*_min_. We could not reproduce our results without averaging over an ensemble of *B* = 50 simulated PIN datasets during burn-in, nor without tempering of *ɛ* and Σ as described in Materials and Methods. Based on our theoretical considerations with smd(*θ*) and cv(*θ*) and our test results, we believe approximation (Equation 4) has been achieved, but note that ultimate evidence cannot be provided since evaluating the likelihood is not feasible to date.

**Table 1:**
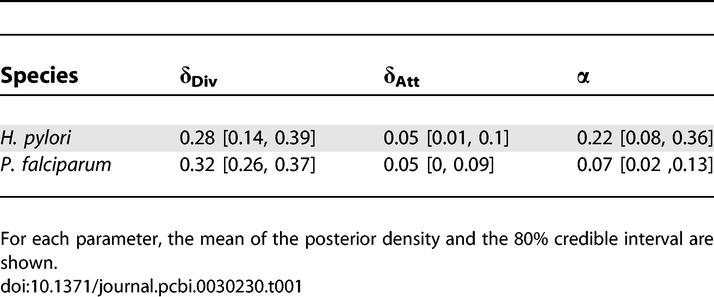
Comparison of the Evolutionary Dynamics Inferred from H. pylori and P. falciparum PIN Data, with LFI Based on WR + DIA + CC + 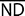 + FRAG

We repeated the LFI analysis on the P. falciparum PIN with the same set of summaries; importantly, these capture global aspects of PIN data simultaneously. The posterior distribution of *θ* for P. falciparum is summarized in Table 1. Notably, the DDa component obtained more weight in the posterior mixture model DDa + PA relative to H. pylori. This suggests, first, that duplication–divergence shapes the global structure of protein networks in a way distinguishable from preferential attachment, and that the difference is also evident when incompleteness of present PIN data is taken into account. Second, gene duplication and interaction divergence might play a larger role in eukaryotic than in prokaryotic protein network evolution, pointing to either discontinuous (i.e., likely to be adaptive) or continuous (i.e., unlikely to be adaptive) taxonomical differences, as already suggested from the extent [42], the size [43,44], and the complexity [45] of protein families.

We found that the lower 80% quantile of 1 − α is larger than 0.6 in both investigated species. Genomic and expression data indicate that repeated single gene duplications with immediate subfunctionalization are a driving force in the evolution of higher organisms [10,11,32,46,47]. Since, on average, DDa mimics duplication with subfunctionalization (see also Box 1), our results emphasize the potential importance of single gene duplications with immediate subfunctionalization in the evolution of the eukaryote. Moreover, we prove in Protocol S1 that DDa may describe any protein network topology due to complementary, random interaction divergence. The precise mechanisms of evolution are less clear for the prokaryote; in particular it is unclear whether horizontal gene transfer is adequately modelled with PA [48], and we caution against interpreting DDa + PA as a model of vertical versus horizontal gene transfer. Nevertheless, the prevalence of duplication divergence in prokaryotic evolution is also indicated from the protein repertoire itself [5,49,50]. In particular, the phylogenetic distributions of protein families over 41 bacteria are consistent with our findings: 60% of protein families in these prokaryotes can be explained by gene duplications alone [50].

The role of duplication divergence in evolution of protein networks across domains we promote here must be considered within the limits of our model and the data. However, we note that our analysis is based on several global features of the network data, which are more reliable than local aspects (Figure S4). More importantly, LFI allows us to take the stochasticity of the evolutionary process and the incompleteness of available network data into account. Also, the credible intervals of *δ*_Div_ and *δ*_Att_ for the P. falciparum PIN overlap with parameter estimates obtained from sequence data of S. cerevisiae. The study of Wagner [37] indicates a mean divergence probability around 35%–42% and a mean attachment probability around 1%–2% within the first 25 million years after a duplication event in this species. Given the number of limitations in both approaches, further work will be required to combine genomic with network data for a detailed reconstruction of the evolution of complex cellular units. Importantly, fitting a model of network evolution that includes link turnover as a case of neofunctionalization might put our conclusions into perspective.

### Inference on Networks Is Consistent and Reliable Only When Summaries Are Combined

**Figure 4:**
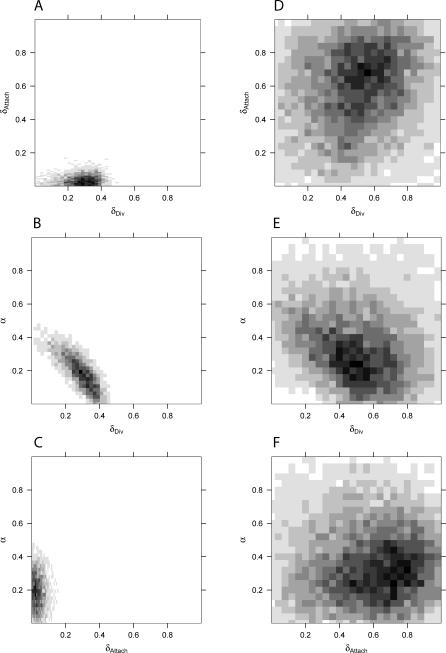
Comparison of Inference with LFI Using One versus Four Summaries for the H. pylori PIN Data (A–C) The 2D histograms of the posterior parameters to the H. pylori PIN dataset, obtained from LFI based on WR + DIA + CC + 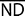 + FRAG. Posterior mass clearly centers on a tight cloud in parameter space. (D–F) For comparison, we ran LFI based on ND alone, adjusted to yield a similar empirical acceptance probability. Although *ɛ*_min_ could be chosen stringently, the 2D histograms are diffuse. The regions of highest posterior density of LFI using ND are inconsistent with those of LFI using WR + DIA + CC + 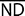 + FRAG.

The complexity of PIN data suggests that LFI on biological network data may be highly influenced by the choice of summaries. Table 2 summarizes that for different combinations of four or more summaries, the respective posterior means and 80% credible intervals coincided with those obtained by WR + DIA + CC + 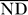 + FRAG. Thus, based on many aspects of PINs, inference on *θ* was consistent. Based on the H. pylori PIN, we found that the approximate posterior (Equation 4) was not identifiable from single summary statistics. Using ND only, it is possible to choose *ɛ*_min_ small, *ɛ*_min_ ≤ 0.35; but Table 2 shows that the inferred 80% credible interval on *θ* is very wide. Considering the parameters *δ*_Div_, *δ*_Att_, and α pairwise, as in Figure 4, illustrates that ND alone leads to two-dimensional high-density regions that are inconsistent with those obtained by four or more summaries. Similarly, LFI based on several other single summary statistics allowed small threshold values *ɛ*_min_, but did not lead to a reliable and consistent estimation of *θ* (unpublished data). This indicates that many evolutionary histories may explain single aspects of PINs almost perfectly without representing the full topology, reflecting the complex nature of biological network data. Our findings relating to ND are particularly worrisome because the degree sequence is a standard descriptor of protein networks, and often kept fixed when generating randomized networks for a significance analysis on aspects of PIN data [28,51,52].

**Table 2:**
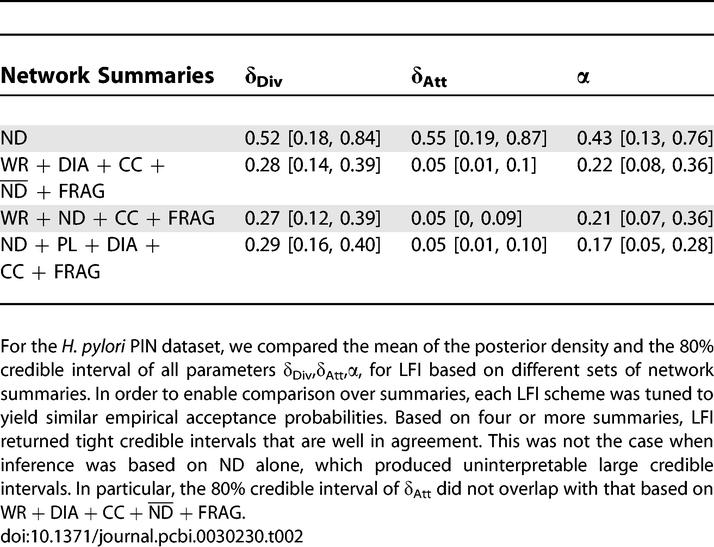
Sensitivity of LFI Based on Different Sets of Summaries

PA alone generates tree-like networks, whereas DDa occasionally produces triangles. Surprisingly, LFI with TRIA included in the set of summary statistics did not aid inference in that convergence took longer and fewer samples were accepted without tightening the credible intervals. Taken together with the fact that other motif counts have a similar high variation over the evolutionary history (unpublished data), this suggests that the extreme variability of motif counts in simulated data reduces their usefulness for inference on biological network data.

### Simple Estimators of the Network Size Are Consistent with LFI Results

**Figure 5:**
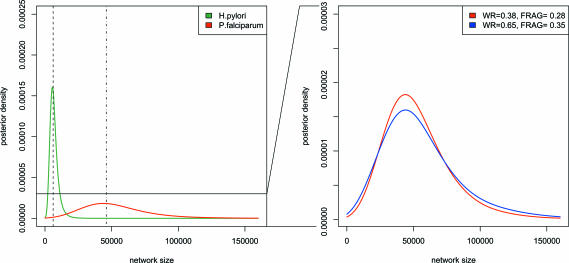
Posterior Densities of the Predicted Network Size for the Complete *H. pylori* and *P. falciparum* PINs with LFI Based on WR + DIA + CC + 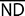 + FRAG (Left) posterior modes (5,636 and 43,835, dashed line and dot-dashed line, respectively) were consistent with the estimator presented in [22] (6,082 and 45,940, respectively; black horizontal lines). The 80% credible interval of the predicted network size for the H. pylori PIN was [2,915, ,536], and the one for the P. falciparum PIN was [18,689, 84,205], illustrating the high variability in the posterior estimate, in particular when the sampling fraction is low (*ρ* = 0.45 and 0.24, respectively). (Right) for the P. falciparum PIN, LFI was repeated using the same set of summaries at relaxed threshold values as indicated in the legend. For display purposes, the *y*-axis was magnified relative to the left figure. As expected, larger thresholds yielded less-confident approximations (Equation 4).

Aspects of the complete, unobserved PINs are easily predicted from the observed networks, once MCMC output is available. Here, we discuss the true network size *R*, by means of its posterior predictive distribution; as outlined in Materials and Methods. The posterior predictive distribution of *R* for H. pylori and P. falciparum is displayed in Figure 5. De Silva et al. [22] proposed a simple estimator of the network size based on the sampling fraction *ρ* of proteins that are present in the dataset. Applied to H. pylori (P. falciparum), the estimate is *R′* = 5,636 (43,835). This is consistent with the posterior predictive distribution obtained by LFI based on WR + DIA + CC + 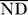 + FRAG in the sense that *Pr*(½ ≤ *R*/*R*′ ≤ 2| D℘) ≥ 0.80.

### Incompleteness Effects Do Not Dominate Evolutionary Network Inference

The fact that current PINs are largely incomplete hampers inference [22,53]. Within our Bayesian framework, we compared the effect of different network order and different levels of incompleteness of PIN datasets on protein network inference (H. pylori: 675, *ρ* = 0.45; and P. falciparum: 1,271, *ρ* = 0.24).

We found large variability associated with predictions of the true network size (see Figure 5); notably, the P. falciparum posterior network size was more diffuse than the one of H. pylori. In order to see whether the large variability arises from the approximative nature of LFI, we repeated LFI based on WR + DIA + CC + 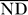 + FRAG for relaxed choices of *ɛ*_min_. Figure 5 shows that tightening the threshold values results in more reliable predictions, and that this effect is negligible when twice as much network data are available. This suggests that aspects of the structure of the true networks remain highly uncertain under the model (Equation 1) when incompleteness is large.

Instead, the credible intervals of all evolution parameters *θ* are tighter for P. falciparum than for H. pylori, even though our model accounts for incompleteness. This indicates that the power of LFI to uncover the evolutionary history of PIN datasets increases with network order irrespective of the levels of incompleteness, essentially because the resolution of the network summaries increases.

**Figure 6:**
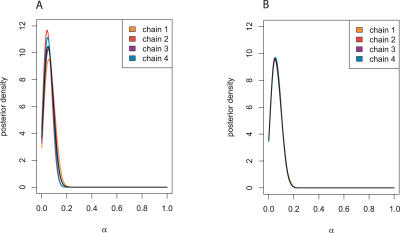
The Effect of Increasing Incompleteness on Summaries For increasingly incomplete PIN datasets of P. falciparum, four MCMC chains were run for 75,000 iterations according to LFI based on WR + DIA + CC + 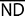 + FRAG. We present the marginal posterior densities of the mixture parameter *α* for two PIN datasets: (A) LFI on four random subsets of order 900 for *ρ* = 0.17 of the P. falciparum PIN dataset (each corresponding to one Markov chain), and (B) LFI on the full P. falciparum PIN dataset for *ρ* = 0.24. The chains were tempered to the minimal threshold values before iteration 800, and converged well onto posterior support. After iteration 800, the chains were taken to represent samples from the posterior, which produced the displayed kernel density estimate. Although LFI is sensitive to the randomly withheld data points, the estimated posteriors of each chain in (A) largely agree with the posterior on the full dataset. This indicates that randomly omitting 500 proteins does not seriously affect algorithm LFI.

We further analysed how the degree of incompleteness affects LFI by randomly withholding more network data of the P. falciparum PIN (*ρ =* 0.17, 0.12, 0.06); see Materials and Methods for details. Briefly, for PINs with *ρ* ≥ 0.17, LFI using WR + DIA + CC + 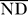 + FRAG was possible, and the parameters were distinguishable in terms of the errors between the real and associated simulated summaries. Table 3 summarizes the 80% credible intervals of all parameters for LFI based on WR + DIA + CC + 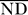 + FRAG for different *ρ*. As expected, highly increased incompleteness implied larger credible intervals. More importantly, randomly omitting 500 proteins from the available PIN of 1,271 proteins did not significantly affect LFI. This is further illustrated with the posterior densities of the mixture parameter *α*, Figure 6.

**Table 3:**
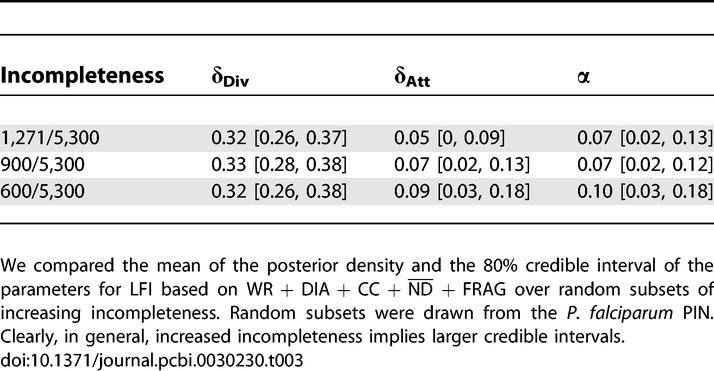
Sensitivity of LFI for PIN Data of Increasing Incompleteness

### Conclusions

PINs from different species have attracted much attention in molecular systems biology. Apart from their suspected role in modulating and underpinning the molecular machinery of complex phenotypes, their evolutionary properties are increasingly being investigated using a range of evolutionary and statistical approaches. We showed that it is possible to draw evolutionary inferences from large-scale, incomplete network data when models of randomly growing graphs are conditioned on many, carefully chosen aspects of the networks. Using a likelihood-free approach that relies on comparing summaries of real network data to simulated PINs, we were able to study more complex models of network evolution at increased confidence than had previously been possible [19].

Our results have important implications for the analysis of protein network topology. Due to its elusive complexity, the topology of a PIN is commonly summarized by the degree sequence [18], as well as the frequency profile of motif counts [28]. An extensive sensitive analysis showed that the degree sequence has very little power to distinguish among different parameters relative to other statistics (Figures 1B and S4B), and fails to infer the parameters correctly (Figure 4). We found that the number of triangles is extremely variable over the evolutionary history of simulated PINs (Figures S1B and S4A) and did not help inference, suggesting that motif counts are risky descriptors of PINs. Instead, if four or more network summaries are combined, then our method yields (i) consistent estimates as well as tight credible intervals on biological data, and (ii) accurate estimates on simulated test data where, by definition, the model is correct. The fact that a reliable, consistent analysis requires the combination of several summaries that capture global aspects of the networks, of which WR is computationally very expensive, renders an implementation targeting the S. cerevisiae PIN dataset extremely challenging.

We used our computational inference scheme to estimate the potential role of aspects of duplication divergence in different domains from large-scale biological network data of H. pylori and P. falciparum, complementing a number of efforts to uncover the mechanisms that underlie the evolutionary history of complex organisms from sequence data [1–3], protein structures [4], or gene families within a wider context [54]. Here, the evolutionary history of PINs was modelled with a mixture of randomly growing graphs that (i) agrees in particular with evolution by single gene duplications and immediate divergence, and (ii) puts minimal assumptions on the time of evolutionary events, because these are difficult to estimate reliably. Crucially, our approach fully deals with incomplete network data and the stochasticity of the underlying evolutionary process. Inference of the evolutionary parameters improves with an increasing order of the PIN data, irrespective of the levels of incompleteness (Figure 6 and Table 3). Within the limits of our evolutionary model and the available data, gene duplication and interaction divergence appear to play a dominant, distinguishably larger part in the evolution of the protein network of the eukaryote P. falciparum (Table 1). Our results emphasize the potential importance of duplication divergence in the evolution of networks across domains. Based on our sensitivity analysis of network summaries, our study suggests, in line with two other recent studies [55,56], that more information could be inferred from combining global aspects of interaction networks than is presently appreciated.

 The opportunities arising from LFI to computational statistics on complex systems are large. Our results emphasize that choosing a set of appropriate summaries is central to maintaining the approximate character of LFI. We proposed the standardized mean derivative and measures of scaled variation to compare the power of summaries one by one. Although ABC-MCMC failed on network data, algorithm LFI enabled efficient and consistent inference. LFI might prove useful in other biological contexts when prior information is relatively vague, and when the underlying model is complex and highly stochastic.

**Figure 7:**
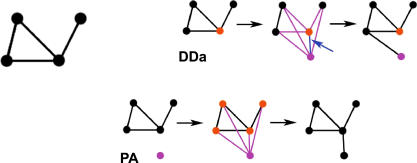
Randomly Growing Graphs

## Materials and Methods

### Algorithm LFI.

For clarity of exposition, we first outline algorithm ABC-MCMC [25] and then present algorithm LFI, which achieves approximation (Equation 4) in protein network inference. Let S℘ = {S_1_,…, S_k_…S_K_} be the chosen set of summary statistics, and let *ɛ* > 0 be a threshold value. Let S℘*_ D℘_*, respectively S℘*_θ_*, denote the set of summary statistics calculated on the observed network D℘, respectively a network simulated with parameter *θ*, and choose some initial parameter value. Then do the following:

**ABC-MCMC1** If now at *θ*, propose a move to *θ′* according to a proposal density *q*(*θ → θ′*).

**ABC-MCMC2** Generate a dataset from *θ′* and compute S℘*_θ′_*.

**ABC-MCMC3** Calculate

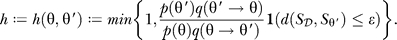



Here, *d*( S℘_ D℘_, S℘*_θ′_*) ≤ *ɛ* denotes that the distance between the *k*th observed and simulated summary statistics is less than *ɛ* for all *k* [23]. The summaries are standardized over the values of the sampled summaries. Different choices of *d* are possible [24]. Here, **1** denotes the indicator function.

**ABC-MCMC4** Accept *θ′* with probability *h* and otherwise stay at *θ*, then return to ABC-MCMC1.

ABC-MCMC is guaranteed to eventually sample from *p*(*θ*|*d*( S℘_ D℘,_ S℘*_θ_*) ≤ ɛ), [25]. We now present algorithm LFI. Let *ɛ_t_* = {*ɛ*_1,*t*_, …, *ɛ_k,t_,* …, *ɛ_K,t_*} be the vector of threshold values at iteration *t*, one for each summary statistic, and let *ɛ_k,_*_min_ be the final, preset threshold value for the *k*th summary statistic after cooling. Similarly, let Σ*_t_* be the variance of the proposal density at iteration *t*, and let Σ_min_ be the final, preset variance after cooling.

**LFI0** If *ɛ_k,t_ ≥ ɛ_k_*_,min_, update *ɛ_k,t_*; if Σ*_t_* ≥ Σ_min_, update Σ*_t_*.

**LFI1** If now at *θ*, propose a move to *θ′* according to a Gaussian density, centred at *θ* with diagonal covariance matrix Σ*_t_* and restricted to the interval [0,1], i.e., *q_t_*(*θ→θ′*) ∝ N(*θ*, Σ*_t_*)**1**_[0,1]_, appropriately normalized.

**LFI2** During the preset, empirically determined burn-in phase, go to LFI2′. Else, generate *B* = 1 PIN according to the mixture model (Equation 1) with parameter *θ′* and grown to the number of open reading frames in the genome of the observed PIN. Take a subnetwork of order that equals the order of the observed PIN. Compute the summaries, put *s_k,θ′_* := *S_k,θ′_* for all *S_k_* ∈ S℘ and go to LFI3.

**LFI2′** Perform LFI2 with *B* = 50 and compute the sample mean *S̄_k_*_,θ_ for all *S_k_* ∈ S℘; in the case of ND and WR, compute the pointwise sample mean. Put *s_k,θ′_*: *= S̄_k_*_,θ_ and go to LFI3.

**LFI3** Calculate

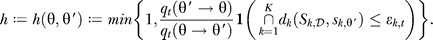



In our case, the prior is uniform, and *p*(*θ′*)/*p*(*θ*) is one. The distance function *d_k_* for the *k*th summary statistic may depend on *k* (see below).

**LFI4** Accept *θ′* with probability *h* and otherwise stay at *θ*, then return to LFI0.

LFI fulfils the detailed balance equations for the same reasons as [25], and hence is guaranteed to eventually sample from

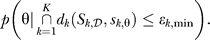



Tempering scheme. We temper the acceptance threshold *ɛ_t_* with an exponential cooling scheme, starting at some initial temperature *ɛ*_0_ and cooling at the next iteration to *ɛ_t_*_+1_ = *γɛ_t_*, until a minimal temperature *ɛ*_min_ is reached. In all cases, the minimal temperature is reached in about 750 iterations. Tempering reduces the number of accepted parameters as the number of iterations increases. We employ a similar exponential cooling scheme on Σ*_t_*, in which the minimal temperature is reached in about 800 iterations. In practice, convergence depends on suitable tempering; we chose *ɛ*_min_ and *γ* for all summary statistics, such that the empirical acceptance probabilities were not too low, and such that the Gelman-Rubin (GR) statistic was well below 1.2 [41], as further detailed in Protocol S1.

*Choice of distance function d*. Our distance function in LFI3 is inspired by the Chebyshev distance proposed in [23] (and outlined in ABC-MCMC3). Notably, since the reliability of PIN summaries differs largely, we combine and do not standardize the summaries; our approach requires *K* different tempering schemes.

For ND and WR, *d_k_* is chosen to capture major pointwise differences in the summaries. Given *S* *_k, D℘_* and *S_k,θ′_* (or *S̄_k_*_,θ′_), we compute the common node degrees (or distances), and for these values, sum the absolute differences of the associated frequencies, cutting off the tails of these distributions.

Initial values. One approach to investigate whether a Markov chain has not yet converged is to start multiple chains at overdispersed initial values. We have started four Markov chains at the initial values (0.9, 0, 0), (0.7, 0.13, 0.23), (0.5, 0.26, 0.46), and (0.3, 0.4, 0.7). The first and the latter initial values represent unrealistic models to check that the chains move toward the support of the distribution. The other two initial values interpolate between these two extremes.

### Network summaries and their analysis.

*Within-reach distribution (WR)*. Given a network D℘ and two connected nodes *i* and *j*, consider the shortest path from *i* to *j* as their distance *d*_ D℘_(*i*, *j*). The (random) number *wr^k^*(*i*) of nodes in distance less than or equal *k* from *i* is then *wr^k^*(*i*) ≔ #{*j*|*d*_ D℘_(*i*, *j*) ≤ *k*}, and the WR is defined as

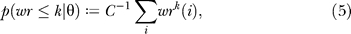
where the normalization constant *C* is the sum of all node pairs in each component in D℘.

*Mean derivative of summary statistics*. In order to analyze the information content of summaries for protein networks, we follow the approach recently proposed by K. Heggland and A. Frigessi [39]. Consider one summary statistic S(*θ,* *G*), evaluated on simulated data *G* generated with parameter *θ*. Heggland and Frigessi argue that “if for fixed *θ*, the variance in S(*θ*,·) is large compared with the derivative of its expectation, it will be more difficult to detect genuine changes at *θ* in S(**·** *,* *G*).” We adopt a variant of their approach, modified to the settings of this paper. Networks *G* *^b^*, *b* = 1, …, 50, are generated for each value of *θ*, and the mean statistics

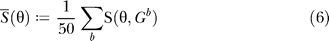
are computed (note that *G* *^b^* is a different realization of the mixture model (Equation 1) for the same values of *θ*). The parameter *θ* has *L* = 3 dimensions, and we integrate over all directional (absolute) mean derivatives to obtain a measure of the overall sensitivity to changes in *θ*:

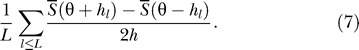



Here, *h* > 0 and *l_D_* is the *L*-dimensional vector that has *h* in dimension *D* and zero otherwise. Since we wish to compare summary statistics, we divide the measure in Equation 7 by the mean of the summary statistic and define the standardized mean derivative:

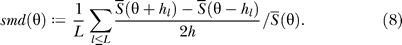



Note that the average cluster coefficient is an observed probability, which is already normalized, and we utilize Equation 7 directly to compute its mean derivative. For the node degree distribution and the WR distribution, we compute the common support of *S̄*(θ + *h_l_*) and *S̄*(θ − *h_l_*), apply Equation 7 pointwise, and sum these values to give smd(*θ*). We chose *h* = 0.025 as an approximation to *h* → 0, which we regard as sufficiently accurate to delineate differences between summary statistics.

*Variation of summary statistics*. Consider a summary statistic S(*θ,* *G* *^b^*) evaluated on simulated data *G* *^b^* generated with parameter *θ*, and the corresponding mean statistic *S̄*(θ) as in Equation 6. We consider the absolute error distribution S(*θ*,*G* *^b^*) − *S̄*(θ), *b* = 1, … , 1,000, scaled appropriately:

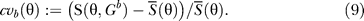



These values yield a relative error histogram for fixed S℘ and *θ*, and we employed the biweight kernel to estimate the density of standardized variation. In the case of CC, ND, and WR, we normalized as detailed above.

### Predicting aspects of PINs.

Aspects or quantities of PINs can be predicted within the Bayesian framework. The posterior predictive distribution of such a quantity, e.g., the network size *R*, may be estimated directly from the MCMC output:

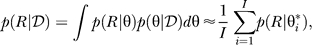
where 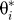 denotes a posterior sample from the set of accepted parameters *θ* after convergence in the MCMC run. We are left to approximate *p*(*R* | 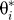 ) by repeatedly generating PINs 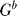 according to 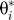 and calculating *R*, i.e.,

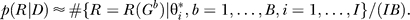



We have chosen *B* = 50 again, and took *I* = 500 samples from the MCMC output.

### Artificially increasing incompleteness of PIN datasets.

Out of 1,271 proteins in the P. falciparum PIN dataset, we randomly chose subgraphs of order *n* = 900, 600, and 300 to mimic increased incompleteness. For each Markov chain in an LFI simulation, such a subgraph was taken as the observed PIN dataset. Consequently, the four chains within one LFI simulation are fitted to slightly varying observations, making inference harder.

## Supporting Information

Figure S1The PIN Datasets Generated by Equation 1 Were Dominated by Stochastic EffectsOne thousand networks to H. pylori (grown to 1,500 nodes and subsampled to 675) are generated with the parameter *θ* = (0.32, 0.02, 0.15), and the squared errors between each summary and the mean summary are recorded. The frequency of cases such that the squared error is greater than values on the abscissa is plotted for WR, 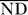 , PL, ND, and TRIA. In 20% of all cases, the squared error in TRIA is greater than 1,000, whereas in all cases, the squared error in ND is not larger than one. Except for ND, large deviations are likely for all summaries, reflecting that stochastic effects dominate network summaries.
(55 KB PDF)

Figure S2 Different Parameters of Equation 1 Leave Distinguishable Imprints on Simulated PINs We compared WR and ND for *α* = 0, 0.2, 1 to the observed summaries of H. pylori (grey) by simulating 50 networks to H. pylori (grown to the number of open reading frames: 1,500, and subsampled to the observed network order: 675) with *θ* = (0.24, 0.04, *α*) for varying *α*. For each within-distance *d* and each node degree *k*, the interquantile range of *p*(*wr* ≤ *d*) and *p*(*k*) for the 50 generated networks was drawn.
(A) The interquantile ranges of WR for PINs generated by different parameters were clearly distinct, and the mixture model with *α* = 0.2 visually improved fit relative to DDa and PA.
(B) On the same scale, the interquantile ranges of ND largely overlapped, indicating that ND might have significantly less power than WR to distinguish between different parameters.
(C) On the log scale for *p*(*k*), the interquantile ranges of ND generated by different parameters were again distinguishable, suggesting that the use of different distance metrics might play an important role in inference on protein network data.
(1.4 MB TIF)

Figure S3Averaging Reduces the Variance of Any Network SummaryMean summaries over larger ensembles of simulated PIN datasets have reduced variance, as exemplified here with DIA. We computed the mean summary (red points) from *B* = 200, 50, 5 networks to H. pylori (grown to 1,500 nodes with *θ* = (0.28, 0.03, 0.21) and subsampled to 675 nodes). In each computation, the 50 networks were randomly chosen from the 200 networks, and then the five networks were randomly chosen from the 50 networks. This procedure was repeated 100 times, and we report the density of the distance of the mean simulated DIA to the observed DIA for *B* = 200, 50, 5. The average of these errors (vertical red line) and the range of one standard deviation (blue) are added. Clearly, the variance of the mean DIA shrinks with increasing *B*, and similarly for all other summaries (unpublished data) with 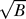 according to the Central Limit Theorem (unpublished data).
(47 KB PDF)

Figure S4 Coefficient of Variation Density across Summaries To compare the variability of the mean posterior summaries of H. pylori, we studied the coefficient of variation density cv(*θ*), described in Materials and Methods, on the grid *θ* ∈ [0.1, 0.7] × [0, 0.5] × [0.1, 0.6] in steps of 0.025. Computations were based on summaries taken from 1,000 simulated PINs to H. pylori (grown to 1,500 nodes and subsampled to 675). We plot the marginal cv(*α*) against *α* for (A) summary statistics and (B) summary distributions. cv complements the information given by smd in Figure 1 to characterize the sensitivity and variability of the summary statistics. TRIA, FRAG, and CC are extremely variable, offsetting their high standardized mean derivatives. ND is almost invariant to random fluctuations and to different parameters. Results for the other two parameters are very similar (unpublished data).
(76 KB PDF)

Protocol S1 Mathematical Properties of the DDa + PA Model of PIN Evolution, Convergence, and LFI on Test Data. (1.1 MB PDF)

## Abbreviations

ABC: approximate Bayesian computation
cv: coefficient of variation density
DDa: duplication divergence with parent–child attachment
LFI: likelihood-free inference
MCMC: Markov Chain Monte Carlo
PA: preferential attachment
PIN: protein interaction network
smd: standardized mean derivative
WR: within-reach distribution
