# Supplementary figures and images for: Using Likelihood-Free Inference to Compare Evolutionary Dynamics of the Protein Networks of H. pylori and P. falciparum
Source: PLoS Comput Biol. 2007 Nov 30;3(11):e230. doi: 10.1371/journal.pcbi.0030230 (PMC2098858; doi:10.1371/journal.pcbi.0030230)

A

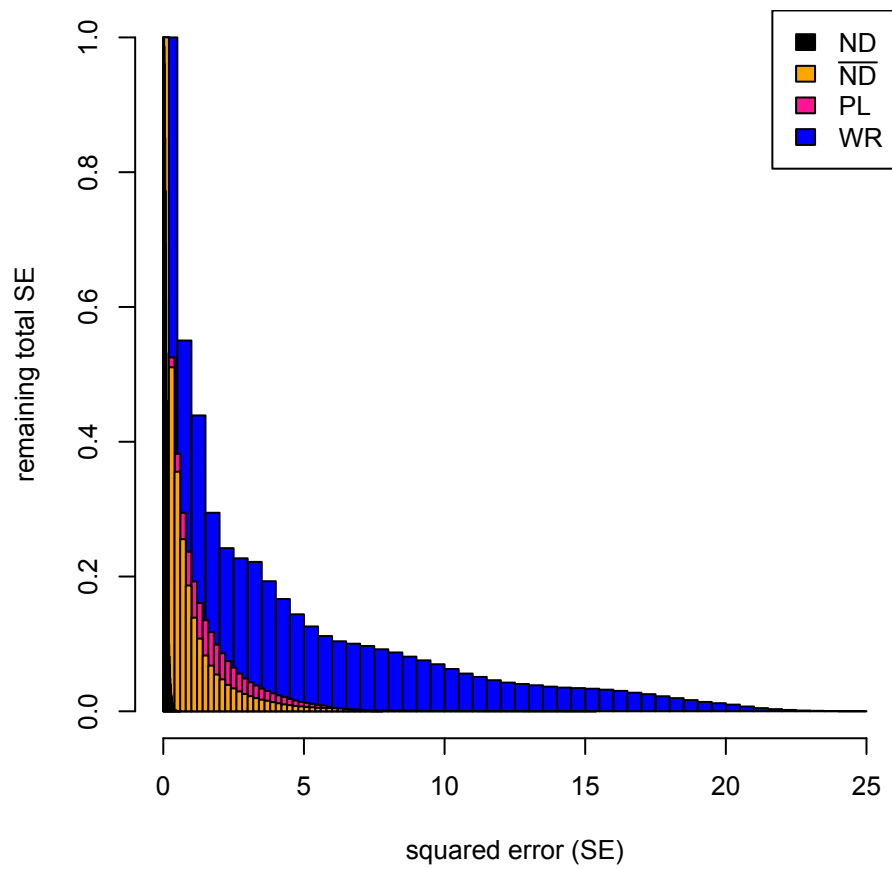

B

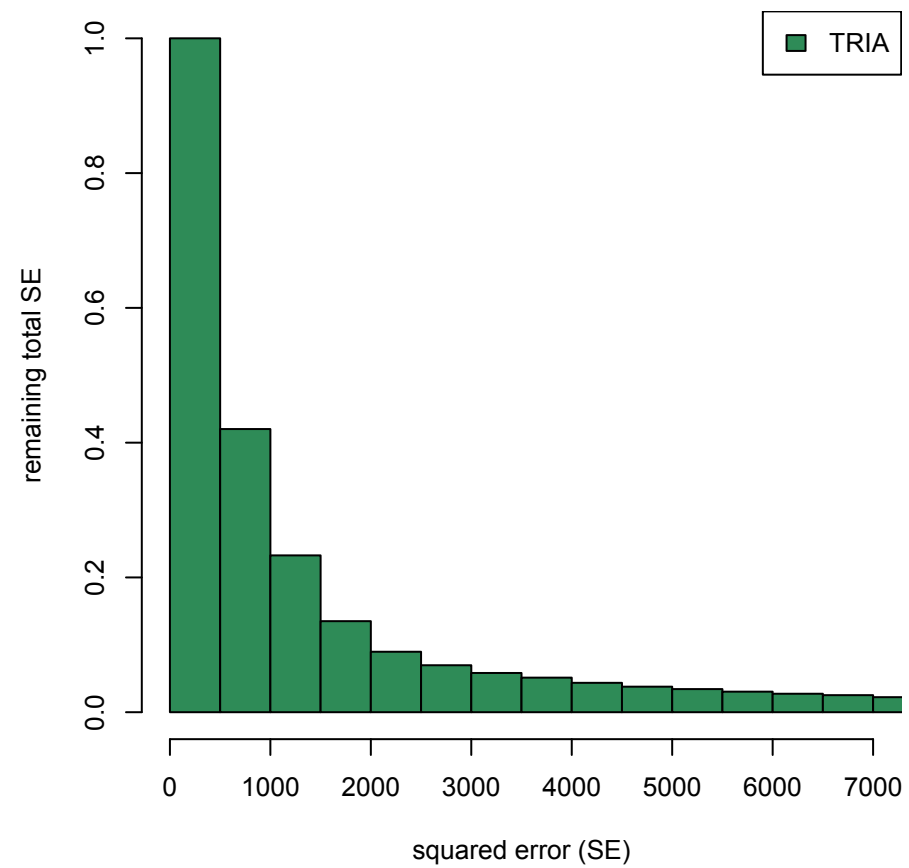

Supplement: Figure S1 — One thousand networks to H. pylori (grown to 1,500 nodes and subsampled to 675) are generated with the parameter θ = (0.32, 0.02, 0.15), and the squared errors between each summary and the mean summary are recorded. The frequency of cases such that the squared error is greater than values on the abscissa is plotted for WR, , PL, ND, and TRIA. In 20% of all cases, the squared error in TRIA is greater than 1,000, whereas in all cases, the squared error in ND is not larger than one. Except for ND, large deviations are likely for all summaries, reflecting that stochastic effects dominate network summaries. (55 KB PDF) [file pcbi.0030230.sg001.pdf]

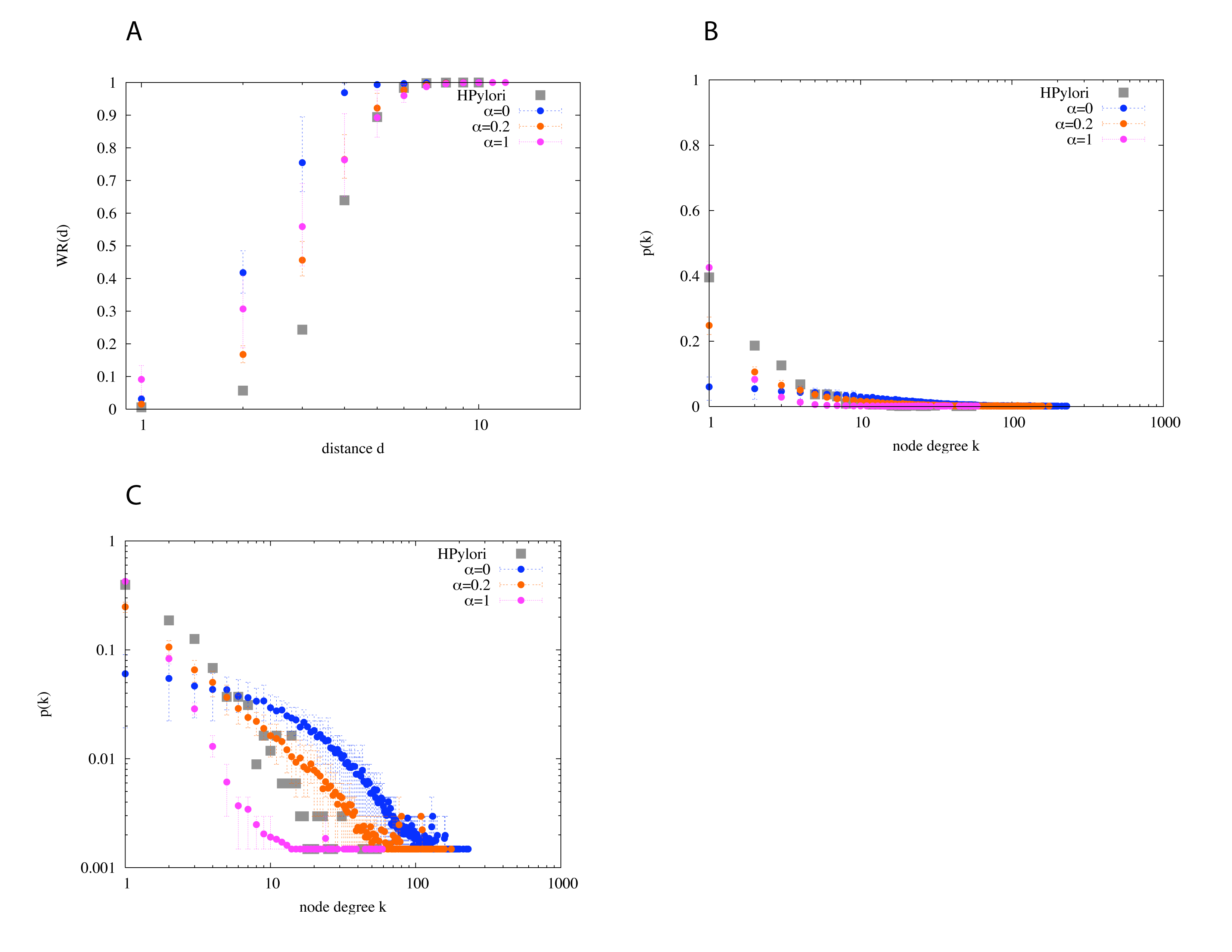

Supplement: Figure S2 — We compared WR and ND for α = 0, 0.2, 1 to the observed summaries of H. pylori (grey) by simulating 50 networks to H. pylori (grown to the number of open reading frames: 1,500, and subsampled to the observed network order: 675) with θ = (0.24, 0.04, α) for varying α. For each within-distance d and each node degree k, the interquantile range of p(wr ≤ d) and p(k) for the 50 generated networks was drawn. (A) The interquantile ranges of WR for PINs generated by different parameters were clearly distinct, and the mixture model with α = 0.2 visually improved fit relative to DDa and PA. (B) On the same scale, the interquantile ranges of ND largely overlapped, indicating that ND might have significantly less power than WR to distinguish between different parameters. (C) On the log scale for p(k), the interquantile ranges of ND generated by different parameters were again distinguishable, suggesting that the use of different distance metrics might play an important role in inference on protein network data. (1.4 MB TIF) [file pcbi.0030230.sg002.tif]

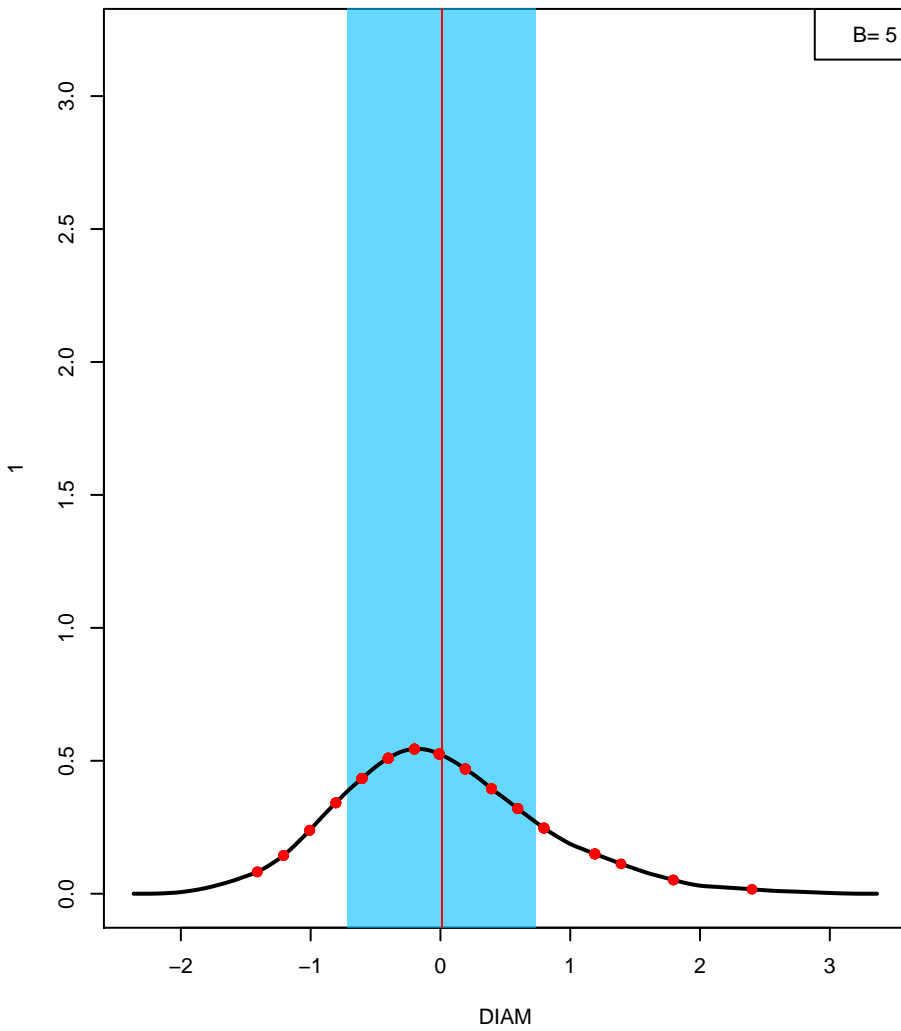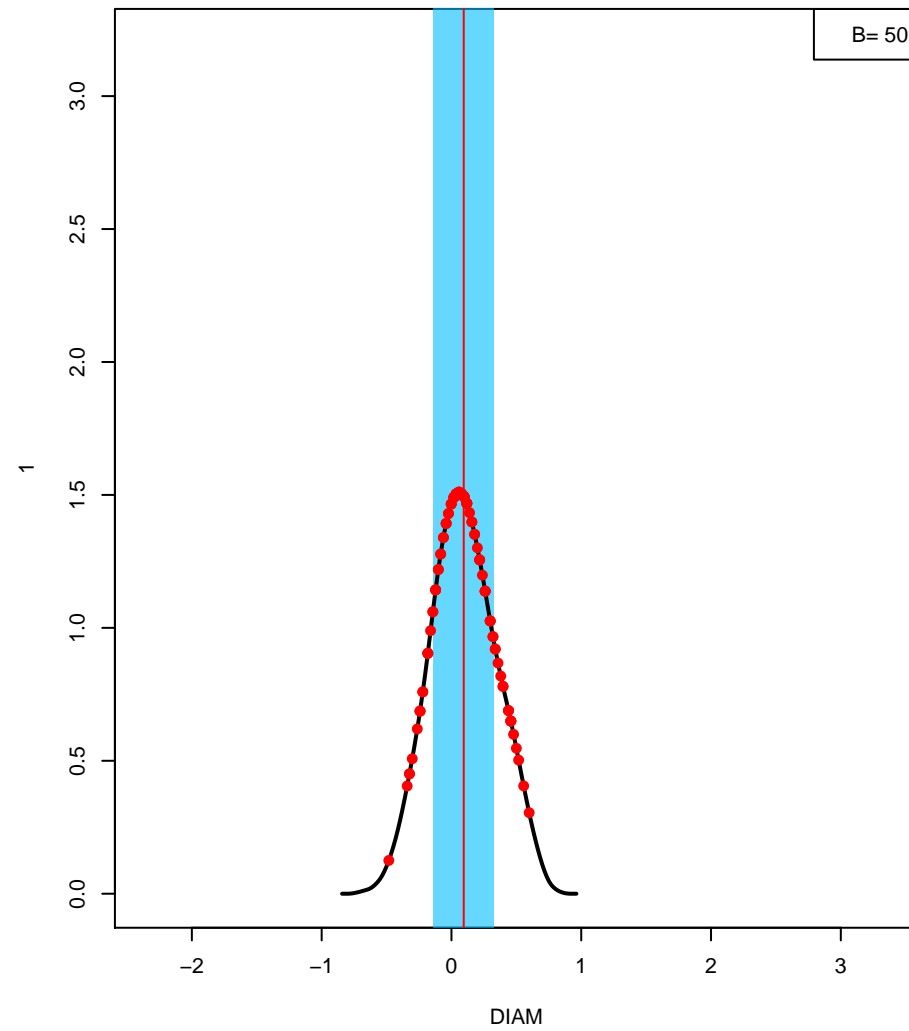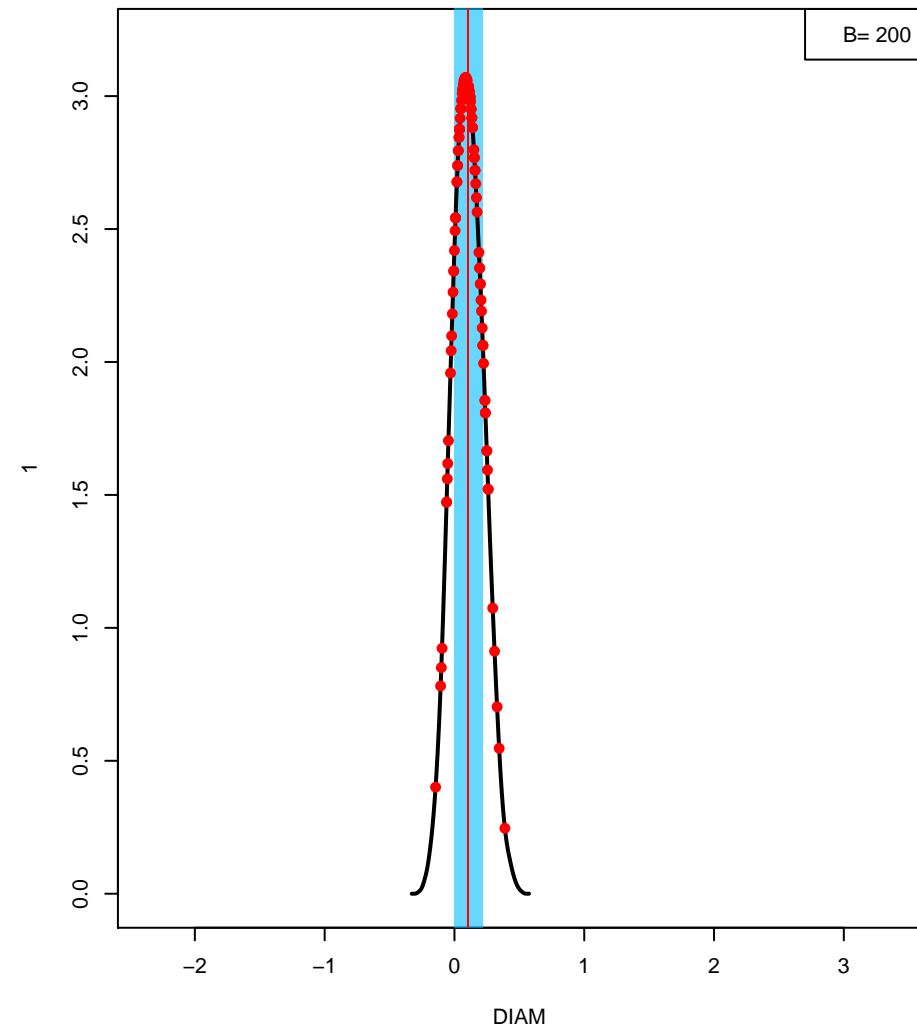

Supplement: Figure S3 — Mean summaries over larger ensembles of simulated PIN datasets have reduced variance, as exemplified here with DIA. We computed the mean summary (red points) from B = 200, 50, 5 networks to H. pylori (grown to 1,500 nodes with θ = (0.28, 0.03, 0.21) and subsampled to 675 nodes). In each computation, the 50 networks were randomly chosen from the 200 networks, and then the five networks were randomly chosen from the 50 networks. This procedure was repeated 100 times, and we report the density of the distance of the mean simulated DIA to the observed DIA for B = 200, 50, 5. The average of these errors (vertical red line) and the range of one standard deviation (blue) are added. Clearly, the variance of the mean DIA shrinks with increasing B, and similarly for all other summaries (unpublished data) with according to the Central Limit Theorem (unpublished data). (47 KB PDF) [file pcbi.0030230.sg003.pdf]

A

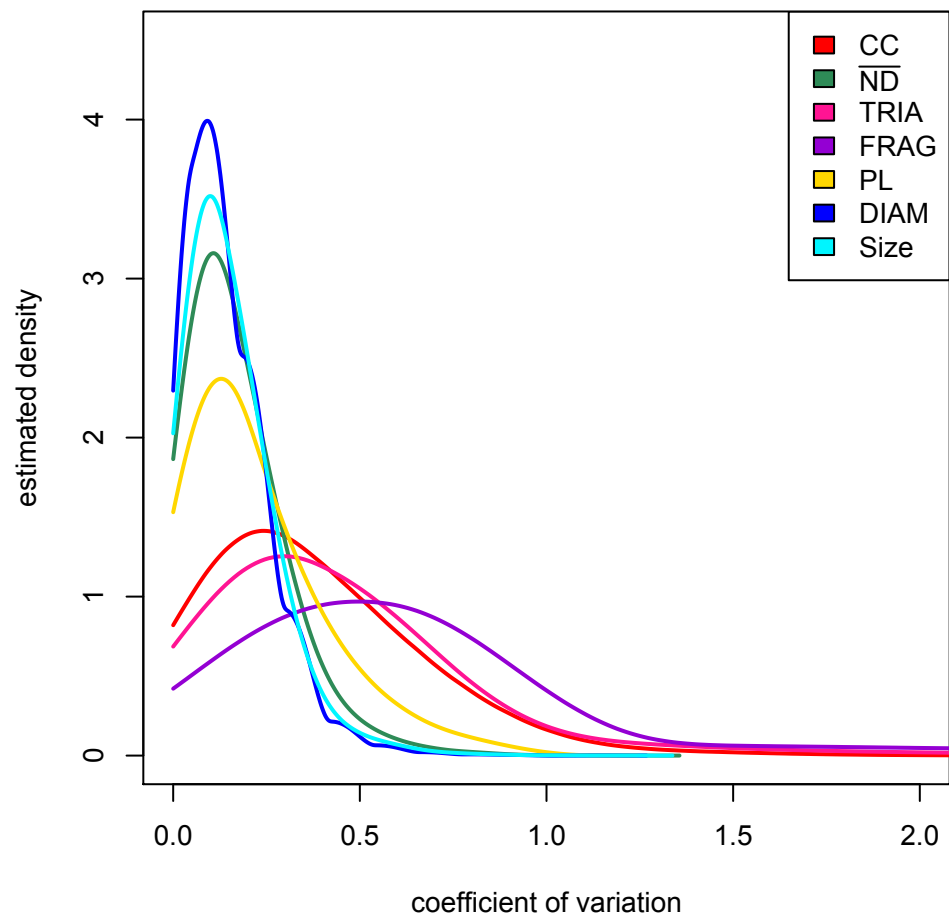

B

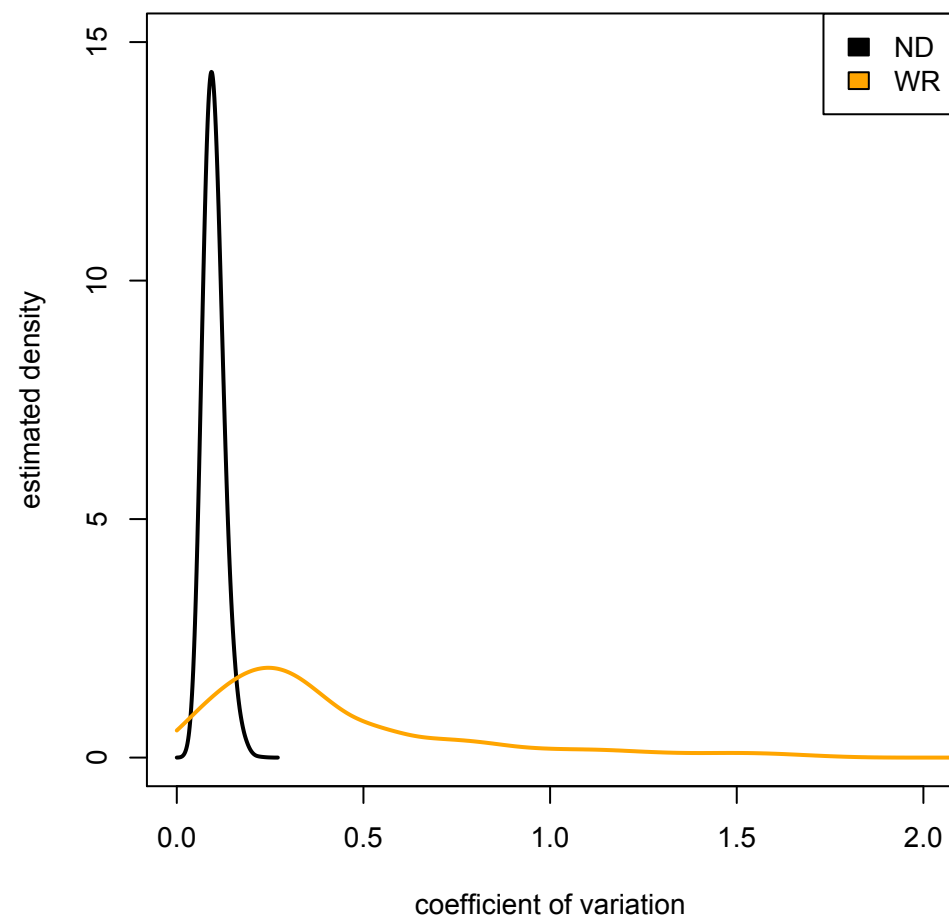

Supplement: Figure S4 — To compare the variability of the mean posterior summaries of H. pylori, we studied the coefficient of variation density cv(θ), described in Materials and Methods, on the grid θ ∈ [0.1, 0.7] × [0, 0.5] × [0.1, 0.6] in steps of 0.025. Computations were based on summaries taken from 1,000 simulated PINs to H. pylori (grown to 1,500 nodes and subsampled to 675). We plot the marginal cv(α) against α for (A) summary statistics and (B) summary distributions. cv complements the information given by smd in Figure 1 to characterize the sensitivity and variability of the summary statistics. TRIA, FRAG, and CC are extremely variable, offsetting their high standardized mean derivatives. ND is almost invariant to random fluctuations and to different parameters. Results for the other two parameters are very similar (unpublished data). (76 KB PDF) [file pcbi.0030230.sg004.pdf]
