# Supplementary material for: Using Likelihood-Free Inference to Compare Evolutionary Dynamics of the Protein Networks of H. pylori and P. falciparum
Source: PLoS Comput Biol. 2007 Nov 30;3(11):e230. doi: 10.1371/journal.pcbi.0030230 (PMC2098858; doi:10.1371/journal.pcbi.0030230)
Supplement: Protocol S1 — (1.1 MB PDF) [file pcbi.0030230.sd001.pdf]

Supporting Information

Mathematical Properties of the DDa+PA model of PIN evolution.

We introduce a more rigorous terminology for the mixture models to PIN evolution presented in Materials and Methods, and show in particular that (1) DDa may explain any protein network topology and (2) computing the likelihood of a PIN generated by DDa is computationally infeasible. Here we focus on the evolution process only and do not consider the sampling component of our model that account for incompleteness in observed PINs.

Let $G = \big(V, E\big)$ denote a graph with node set V and edge set E, where $e \in E$ if there are nodes $u, v \in V$ such that e is a link between u and v. We always take G to be undirected without multiple edges, self-loops and labelled.

Definition S.1 *A randomly growing graph is a Markov chain $\big\{G_t\big\}_{t=t_0}^{\infty}$ such that G_t is a subset of the nodes of G_{t+1}. Denote the transition probability from G_t to G_{t+1} by $P(G_{t+1}|G_t)$ and consider the transition kernel $P := P(\cdot|\cdot)$. A randomly growing graph together with a stopping rule T defines an evolution graph $\big\{G_t\big\}_{t=t_0}^{T}$. Equivalently, a transition kernel, an initial graph and a stopping rule uniquely define an evolution graph.*

Note that the above definition readily implies that only one node be added per time. Since graphs are labelled, the last node added in G_t is uniquely determined. Here we consider deterministic stopping rules only. In this text we focus on the transition kernel P that corresponds to evolution dynamics. It is straighforward to determine the transition probabilities for the mixture model presented in the main text; recall in particular that the node u for duplication is chosen uniformly. Denote the growth parameters of this mechanism by $\theta = \big(\delta_{\text{Div}}, \delta_{\text{Att}}, \alpha\big)$.

Lemma S.2 *Given G_t and G_{t+1}, assume that node u is the parent of the new node v in G_{t+1}. We have that*

$$P(G_{t+1}|G_t, u, \theta) = \alpha PA(u, v) + (1 - \alpha) DDa(u, v, \delta_{Div}, \delta_{Att}) \tag{S.10}$$

where $PA(u, v) := \mathbf{1}\big(e(u, v) = 1 \wedge d(v) = 1\big)$,

$$DDa(u, v, \delta_{Div}, \delta_{Att}) := C\big[\delta_{Att}^{e(u,v)}(1 - \delta_{Att})^{1-e(u,v)}\big]\Big(\big[1 - \delta_{Div}\big]^{2N_{11}(u,v)}\big[\delta_{Div}(1 - \delta_{Div})\big]^{N_{10}(u,v)}\Big),$$

$e(u, v)$ is 1 if there is an edge between u and v and 0 otherwise, $d(v)$ denotes the degree of node v, $N_{11}(u, v) := \#N(u) \cap N(v)$ and $N_{10}(u, v) := \#N(u) \cup N(v) - \#N(u) \cap N(v) - e(u, v)$. The constant C of normalization is $(1 - {\delta_{Div}}^2)^{N_{11}(uv)+N_{10}(u,v)}$.

Proof: Suppose that x in G_t duplicated and diverged to the nodes u and v in G_{t+1}. Let $N(x)$ be the set of nodes in G_t that are adjacent to x, and denote for all u,v the set numbers $M_{11}(u, v) := \#\big\{y \in N(x)\big|y \in N(u) \wedge y \in N(v)\big\}$, $M_{01}(u, v) := \#\big\{y \in N(x)\big|y \notin N(u) \wedge y \in N(v)\big\}$ and $M_{10}(uv) := \#\big\{y \in N(x)\big|y \in N(u) \wedge y \notin N(v)\big\}$. Since edges duplicate and diverge uniformly and independently, the transition probability of duplication-divergence discarding links between the parent and child is proportional to

$$\big[(1 - \delta_{\text{Div}})(1 - \delta_{\text{Div}})\big]^{M_{11}(u,v)}\big[\delta_{\text{Div}}(1 - \delta_{\text{Div}})\big]^{M_{01}(u,v)}\big[(1 - \delta_{\text{Div}})\delta_{\text{Div}}\big]^{M_{10}(u,v)}.$$

If we denote $N_{11}(u,v)$ and $N_{10}(u,v)$ as above, then the above expression simplifies to

$$\big[1-\delta_{\text{Div}})\big]^{2N_{11}(u,v)}\big[\delta_{\text{Div}}(1-\delta_{\text{Div}})\big]^{N_{10}(u,v)},$$

and (S.10) follows easily. □

Corollary S.3 *The transition probability from G_t to G_{t+1} given the set θ of growth parameters is*

$$P(G_{t+1}|G_t,\theta)=\frac{1}{t}\sum_u P(G_{t+1}|G_t,u,\theta). \tag{S.11}$$

If not stated otherwise, the (standard) mixture model in this article is the evolution graph with transition probabilities (S.11), initial graph G_0 that connects two nodes with one link and stopping time $T \ll \infty$. For *H. pylori* and *P. falciparum*, we take $T = 1500$ and $T = 5300$ respectively.

Definition S.4 *For a graph G_t and a node v in G_t, denote G_t where node v is removed by $G_t(-v)$. v is said to be removable if $P\big(G_t\big|G_t(-v),\theta\big) > 0$ for some θ. If G_t contains removable nodes, then it is called reducible, otherwise irreducible. An evolution graph is reducible, if $P(G_t|G_1) > 0$ for some θ and any graph G_t, where G_1 is the graph consisting of one node.*

Lemma S.5 *Consider the transition kernel P of the mixture model. For any nontrivial graph G_t and a node v of G_t*

$$P\big(G_t\big|G_t(-v)\big) > 0,$$

where we set δ_{Att} and δ_{Div} different from $0, 1$ and $\alpha < 0$.

Proof: Since P does not produce a graph with nodes of degree 0, we may choose a node $w \neq v$ in G_t. If G_t contains only v and w, then there must be an edge between them and clearly $p\big(G_2\big|G_1,v,\theta\big) = 1-\delta_{\text{Att}} > 0$. Let us now assume that G_t contains more than two nodes. Consider $N := N(v)\cup N(w)\setminus\{v,w\}$. Then, we may construct $G_t(-v)$ as the graph that results after replacing v,w and $N(v)$,$N(w)$ with its common ancestor u,N. □

Applying Lemma S.5 repeatedly, gives the following Corollary.

Corollary S.6 *The mixture model is reducible.*

Corollary S.6 guarantees that the the mixture model can explain any graph G topologically. In particular, Lemma S.5 is based on properties of DDa, and hence a fortiori DDa can explain any graph topologically. It also shows that every node is removable and hence there are $T!$ ways to reduce any graph G_t with DDa. This is significantly less for the transition kernel presented in [19], making the inference scheme therein computationally feasible.

Summarizing aspects of PINs

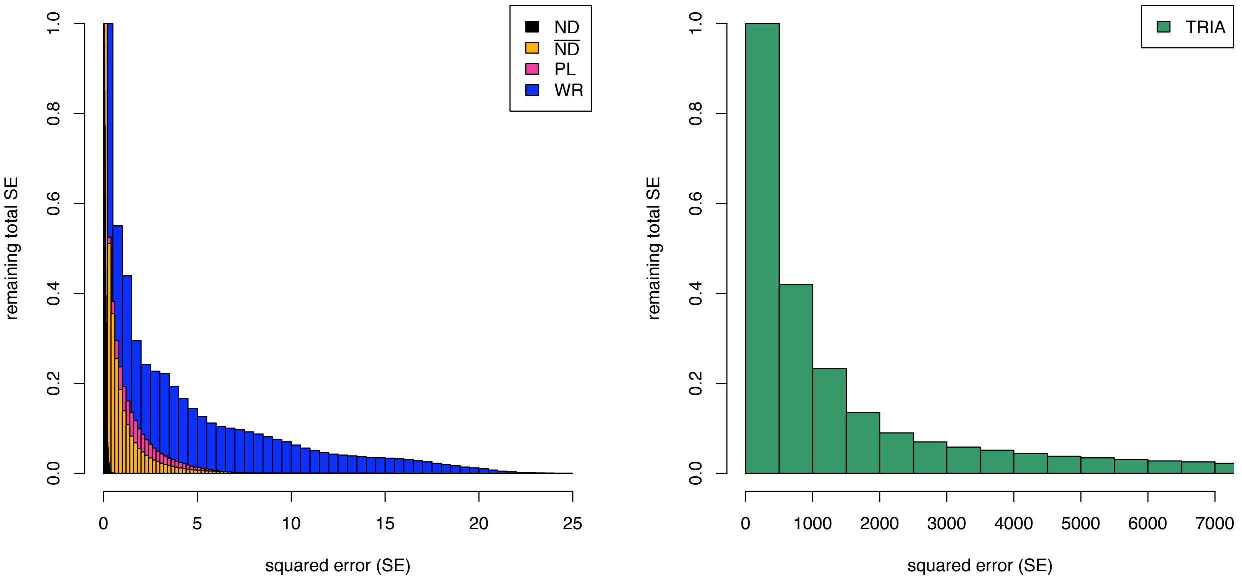

(a) (b)

Figure S.1: The PIN data sets generated by process (1) were dominated by stochastic effects. 1000 networks to *H. pylori* (grown to 1500 nodes and subsampled to 675) are generated with the parameter $\theta = (0.32, 0.02, 0.15)$, and the squared errors between each summary and the mean summary are recorded. The frequency of cases such that the squared error is greater than values on the abscissa is plotted for WR, $\overline{\text{ND}}$, PL, ND and TRIA. In 20% of all cases, the squared error in TRIA is greater than 1000, while in all cases the squared error in ND is not larger than 1. Except for ND, large deviations are likely for all summaries, reflecting that stochastic effects dominate network summaries.

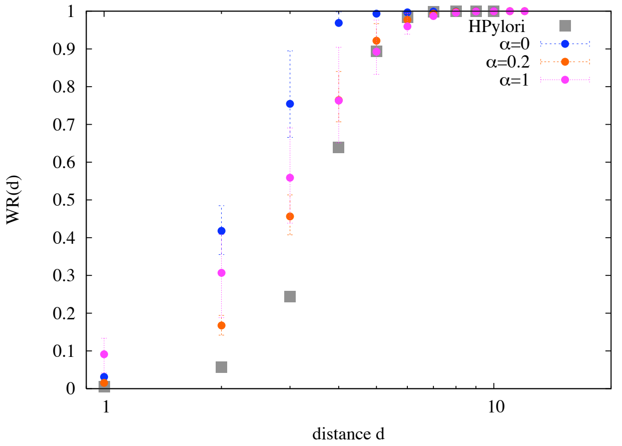

(a)

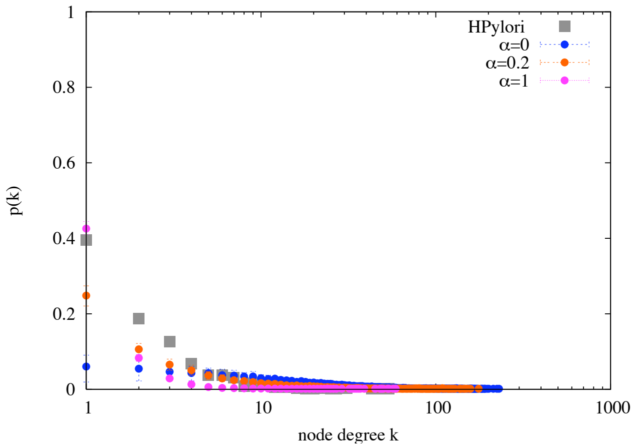

(b)

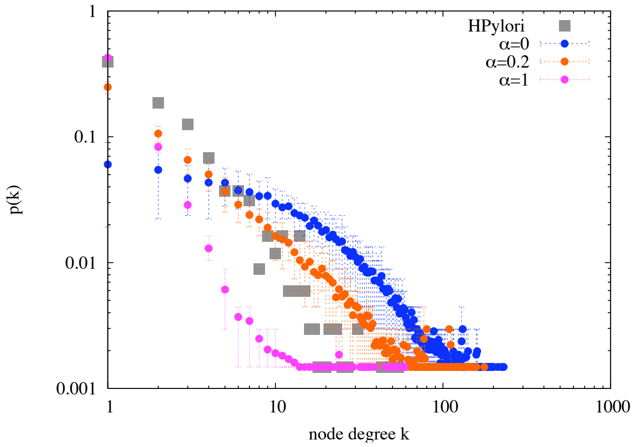

(c)

Figure S.2: Different parameters of model (1) leave distinguishable imprints on simulated PINs. We compared WR and NDfor $\alpha = 0, 0.2, 1$ to the observed summaries of *H. pylori* (grey) by simulating 50 networks to *H. pylori* (grown to the number of open-reading frames, 1500, and subsampled to the observed network order, 675) with $\theta = (0.24, 0.04, \alpha)$ for varying α. For each within-distance d and each node degree k the interquantile range of $p(wr \leq d)$ and $p(k)$ for the 50 generated networks was drawn. (a) The interquantile ranges of WR for PINs generated by different parameters were clearly distinct, and the mixture model with $\alpha = 0.2$ visually improved fit relative to DDa and PA. (b) On the same scale, the interquantile ranges of ND largely overlapped, indicating that ND might have significantly less power than WR to distinguish between different parameters. (c) On the log scale for $p(k)$, the interquantile ranges of ND generated by different parameters were again distinguishable, suggesting that the use of different distance metrics might play an important role in inference on protein network data.

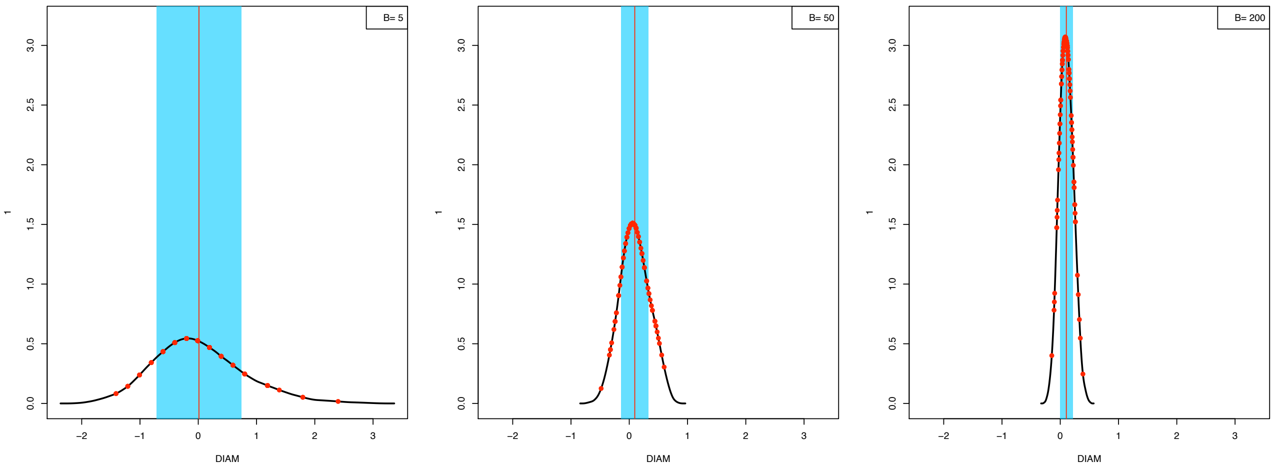

Figure S.3: Mean summaries over larger ensembles of B simulated PIN data sets have reduced variance, as exemplified here with DIAM. We computed the mean summary (red points) from $B = 200, 50, 5$ networks to *H. pylori* (grown to 1500 nodes with $\theta = (0.28, 0.03, 0.21)$ and subsampled to 675 nodes). In each computation, the 50 networks were randomly chosen from the 200 networks and then the 5 networks randomly from the 50 networks. This procedure was repeated 100 times, and we report the density of the distance of the mean simulated DIAM to the observed DIAM for $B = 200, 50, 5$. The average of these errors (vertical red line) and the range of one standard deviation (blue) are added. Clearly, the variance of the mean DIAM shrinks with increasing B, and similarly for all other summaries (not shown) with $\sqrt{B}$ according to the Central Limit Theorem (not shown).

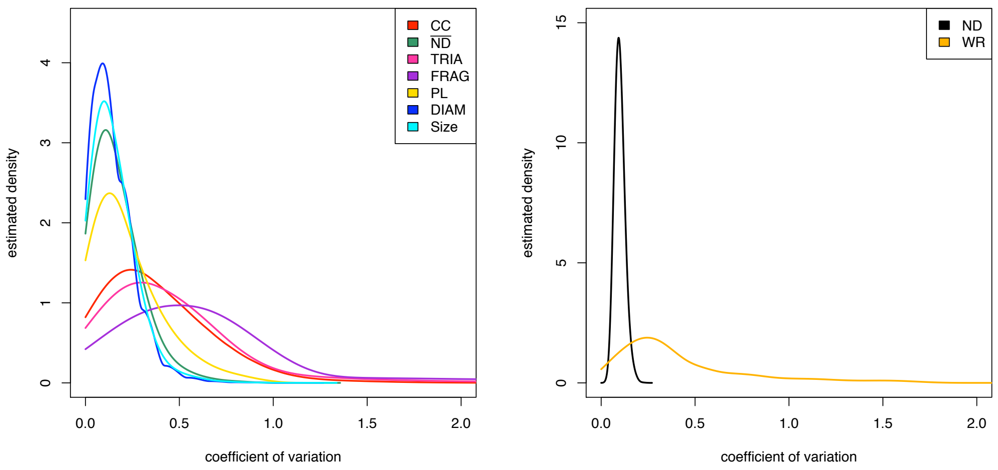

(a) (b)

Figure S.4: To compare the variability of the mean posterior summaries of *H. pylori*, we studied the density of standardized variation cv(θ), described in Materials and Methods, on the grid $\theta \in [0.1, 0.7] \times [0, 0.5] \times [0.1, 0.6]$ in steps of 0.025. Computations were based on summaries taken from 1000 simulated PINs to *H. pylori* (grown to 1500 nodes and subsampled to 675). We plot the marginal cv(α) against α for (a) summary statistics and (b) summary distributions. cv complements the information given by smd in Figure 1 to characterize the sensitivity and variability of the summary statistics. TRIA, FRAG and CC are extremely variable, offsetting their high standardized mean derivatives. ND is almost invariant to random fluctuations and to different parameters. Results for the other two parameters are very similar (not shown).

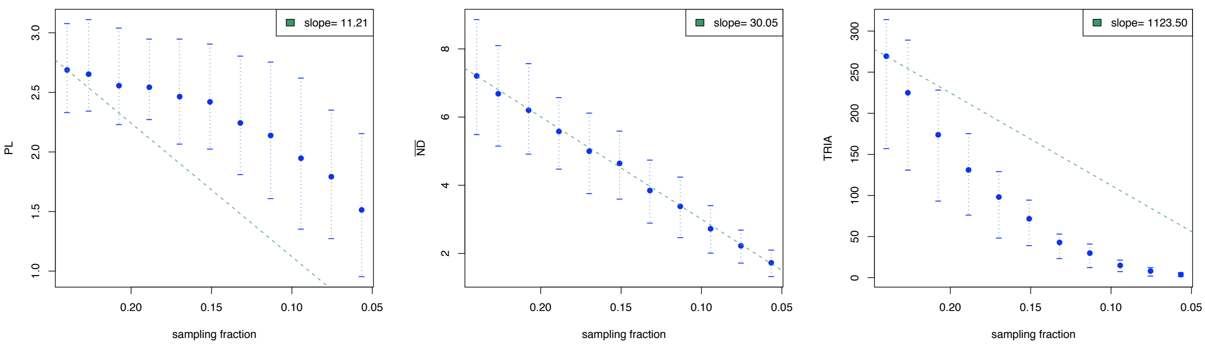

Figure S.5: Many proteins are missing in PINs, and, ideally, summaries should be robust under increasing incompleteness. For a given parameter θ, we compared the curvature of decay of $\overline{\text{ND}}$ with those of PL and TRIA under decreasing ρ. The interquantile range and mean summaries for PL, $\overline{\text{ND}}$ and TRIA are displayed (in blue) as a function of decreasing sampling fraction ρ. Computations were based on 250 networks to *P. falciparum* (grown to 5300 nodes with $\theta = (0.32, 0.04, 0.05)$, and subsampled according to ρ). The green dashed line corresponds to proportional decay relative to the sampling fraction and is computed as follows. Denote the observed sampling fraction of the *P. falciparum* PIN data set with $\rho_{\mathcal{D}} = 0.24$, and a mean simulated summary to an ensemble of simulated PINs at sampling fraction ρ with $\overline{\mathcal{S}}_{\theta,\rho}$. Given another sampling fraction ρ', the slope of the green dashed line is $\big(\overline{\mathcal{S}}_{\theta,\rho_{\mathcal{D}}} - \frac{\rho'}{\rho_{\mathcal{D}}}\overline{\mathcal{S}}_{\theta,\rho_{\mathcal{D}}}\big)/\big(\rho_{\mathcal{D}} - \rho'\big)$. We considered $\rho' = 0.22$; the intercept is computed analogously. Apart from CC, all other summaries were distorted under decreasing ρ. While $\overline{\text{ND}}$ decreased linear to the sampling fraction, PL decreased sublinearly and TRIA superlinearly. This indicates that at low sampling fraction, global aspects of PINs are much less distorted than motif counts in biological network data.

**Network Summaries**	avg. acc. prob.	GR($\boldsymbol{\delta}_{\mathbf{Div}}$)	GR($\boldsymbol{\delta}_{\mathbf{Att}}$)	GR($\boldsymbol{\alpha}$)
ND	0.15	1.00	1.01	1.00
WR+DIA+CC+ $\overline{\text{ND}}$+FRAG	0.02	1.00	1.00	1.00
WR+ND+ CC+FRAG	0.06	1.01	1.01	1.00
ND+PL+ DIA+CC+FRAG	0.03	1.00	1.01	1.01

Table S.1: Convergence analysis of LFI on the *H. pylori* PIN data set. For each set of summaries, the average empirical acceptance probabilities and the GR-statistics are reported for each evolution parameter; see text in the Supporting Information. All runs converge well as indicated by the GR-statistic, and the empirical acceptance probabilities are between $[0.02, 0.15]$ to facilitate cross-comparison.

Convergence analysis of likelihood-free inference.

MCMC is guaranteed to converge in distribution to the posterior under some regularity conditions. The MCMC output may be directly used to determine the burn-in period, at which the Markov chain has not yet converged. After burn-in, MCMC continues and is taken to sample from the posterior. Such analysis is particularly important for likelihood-free inference within MCMC, because of (i) the nontrivial choice of ε in combination with the set of summaries $\mathbb{S}$ and (ii) the nature of the ABC algorithm.

Suppose we start many Markov chains at overdispersed initial parameter values. A standard technique to detect that all chains have not yet converged is to compare the variance within one chain against the variance between all chains [41]: if this ratio exceeds 1.2, then convergence is usually rejected. In this case we argue that the independent chains have not explored the state space sufficiently well, and must run longer.

Tables S.1 and S.2 document the empirical acceptance probabilities and the Gelman-Rubin test for all Markov chain runs presented in the main text. MCMC output is taken to represent posterior samples after 800 iterations if the Gelman-Rubin statistic does not exceed 1.2. This criterion ensures that posterior samples are taken after tempering the MCMC chains.

**Incompleteness**	avg. acc. prob.	GR($\boldsymbol{\delta}_{\mathbf{Div}}$)	GR($\boldsymbol{\delta}_{\mathbf{Att}}$)	GR($\boldsymbol{\alpha}$)
1271/5300	0.04	1.00	1.00	1.00
900/5300	0.03	1.00	1.03	1.02
600/5300	0.02	1.08	1.03	1.06

Table S.2: Convergence analysis of LFI on the *P. falciparum* PIN data set. For PIN data sets of increasing incompleteness, we report the average empirical acceptance probabilities and the GR-statistic for the evolution parameters; see text in the Supporting Information. LFI with $\rho \geq 0.17$ (900/5300) converged very well as indicated by the GR-statistic. With increasing ρ, LFI converged less well.

Preliminary Tests of LFI on simulated protein networks

Accuracy PIN test data sets were generated based on model (1) with the parameter $\theta = (0.4, 0.3, 0.3)$. To simulate incompleteness, we grew the PIN test data sets to 120 proteins and retained a subgraph of 100 proteins. For each test, 4 independent PIN test data sets were generated in this way. We believe that the performed tests were demanding because the protein test networks are (1) very small and (2) highly variable so that characteristics genuine to the evolution mechanism and yet common to all networks are highly diluted. Then we applied the LFI scheme detailed in Materials and Methods, with each Markov chain using a different PIN test data set. We tried a number of different sets of network summaries and studied the accuracy in re-estimating θ. LFI based on WR+DIA+CC+$\overline{\text{ND}}$+FRAG was particularly successful. For example, Figure S.7 compares how accurately LFI based on WR+DIA+CC+$\overline{\text{ND}}$+FRAG and LFI based on ND re-estimate θ in terms of 2D-histograms of the posterior of θ.

Sensitivity For a number of different summaries, we also studied if LFI is sensitive to changes in the evolution model. Similar as above, we generated PIN test data sets according to DDa or PA with the parameters $\theta = (0.4, 0.3, 0)$, and $\theta = (0.4, 0.3, 1)$ respectively. LFI based on WR+DIA+CC+$\overline{\text{ND}}$+FRAG may distinguish between DDa and PA. Figure S.6 possibly illustrates best that the mixture parameter α is in favor of the correct model (orange), and that the marginal posterior of α separate clearly. Similar results were obtained for other tests based on 4 or more network summaries (data not shown).

In summary, our method correctly predicted the true model parameter with accuracy, converged quickly and clearly distinguished between different evolution mechanisms only when 4 or more different summaries with non-zero gradient and moderate variation were used. Notably, ND alone could not re-estimate the true model parameters confidently, while the set of summaries composed of WR+DIA+CC+$\overline{\text{ND}}$+FRAG performed very well relative to other combinations. We could not reproduce similar results without either tempering or smoothing.

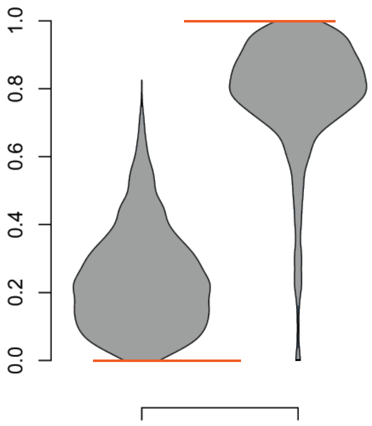

Figure S.6: Violin plot of the marginal posterior of α for LFI based on WR+DIA+CC+$\overline{\text{ND}}$+FRAG on PIN test data sets. (left) The PIN test data sets were generated by $\theta = (0.4, 0.3, 0)$. (right) The PIN test data sets were generated by $\theta = (0.4, 0.3, 1)$. In both cases, the re-estimated marginal posterior of α is in favor of the correct model (orange lines), and the bulk of the marginal posteriors are clearly separated.

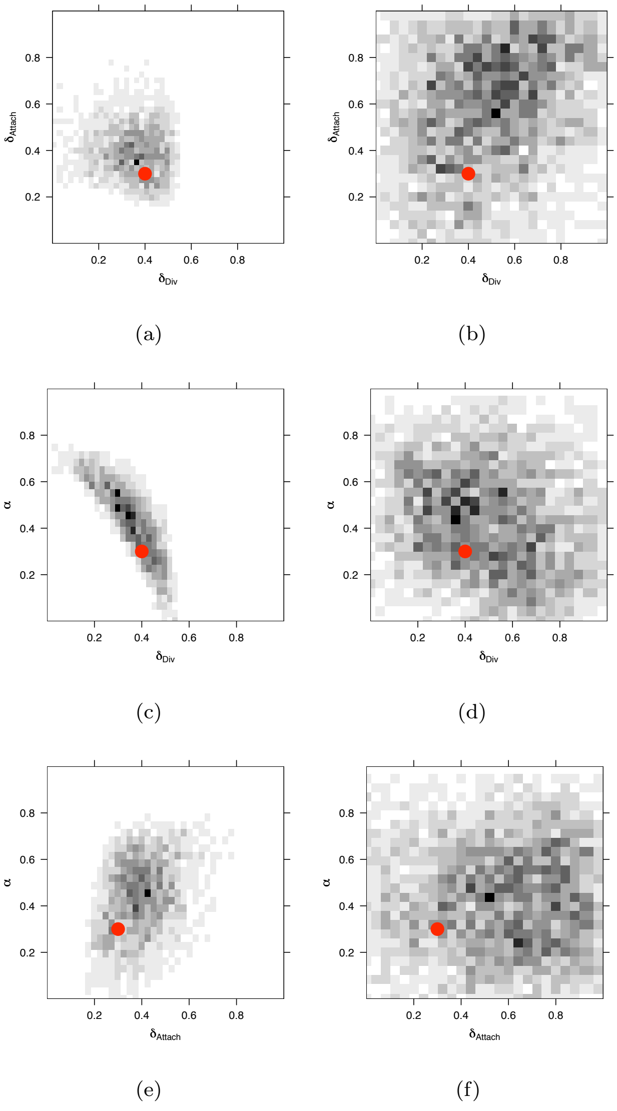

(a) (b) (c) (d) (e) (f)

Figure S.7: LFI to re-estimate the parameter from 4 randomly created test PINs generated with $\theta = (0.4, 0.3, 0.3)$ as described in the Supplementary Information. (a,c,e) 2D-histograms of the posterior parameters with LFI based on WR+DIA+CC+$\overline{\text{ND}}$+FRAG. The posteriors are centered around the true parameter (red). (b,d,f) For comparison, 2D-histograms of the posterior parameters based on LFI with ND. Here, the posteriors appear diffuse and are not centered around the true parameter.

**Network Summaries**	avg. acc. prob.	GR($\boldsymbol{\delta}_{\mathbf{Div}}$)	GR($\boldsymbol{\delta}_{\mathbf{Att}}$)	GR($\boldsymbol{\alpha}$)
ND	0.30	1.02	1.01	1.01
WR+DIA+CC+ $\overline{\text{ND}}$+FRAG	0.31	1.03	1.04	1.10

Table S.3: Convergence analysis for LFI to fit the PIN test data sets, accompanying Figure S.7. For each set of summaries, the average empirical acceptance probabilities and the GR-statistics are reported for each evolution parameter; see text in the Supporting Information. All runs converge as indicated by GR, and the empirical acceptance probabilities are similar, allowing a cross-comparison.
